# Evaluation of *Atractylodes macrocephala* Koidz Extract as a Multi-Functional Natural Ingredient for Skin Barrier Repair

**DOI:** 10.3390/molecules31203713

**Published:** 2026-10-16

**Authors:** Huan Liu, Le Yu, Chang-Yin Yang, Yu-Qiang Zhao, Lin Xu, Ying Zhou

**Affiliations:** 1 Yunnan Characteristic Plant Extraction Laboratory, School of Chemical Science and Technology, Yunnan University, Kunming 650091, China; liuhuan@stu.ynu.edu.cn (H.L.); yule@ynu.edu.cn (L.Y.); yuqiangzhao@ymu.edu.cn (Y.-Q.Z.); 2 Liwa Institute of Skin Health, School of Chemistry and Molecular Engineering, East China Normal University, Shanghai 200062, China; 52274300033@stu.ecnu.edu.cn

**Keywords:** *Atractylodes macrocephala* Koidz, skin barrier, oxidative stress, ceramide synthesis, enzyme inhibition, anti-inflammatory, NF-κB pathway, anti-dermatophyte activity

## Abstract

Epidermal barrier damage triggers sensitive skin disorders. Synthetic skin repair agents frequently incur irritation, whereas medicinal and edible herbal ingredients offer milder alternatives. As a traditional Chinese medicinal herb, *Atractylodes macrocephala* Koidz remains inadequately characterized regarding its epidermal barrier repair efficacy. This work systematically characterized the barrier restoring capacity of *A. macrocephala* extract (**AKE**) via keratinocyte injured models, biochemical assays, spectroscopic interaction analysis, molecular docking and a tape stripping-induced mouse model of skin barrier impairment. **AKE** promoted keratinocyte migration, alleviated sodium lauryl sulfate triggered oxidative injury and enhanced ceramide synthesis. Moreover, **AKE** inhibited hyaluronidase, collagenase and elastase activities. Fluorescence quenching spectroscopy and thermodynamic analysis demonstrated spontaneous binding between **AKE** constituents and these skin degrading enzymes. At 50 μg/mL, **AKE** downregulated expression of pro-inflammatory cytokines interleukin (IL)-6 and IL-8 by suppressing the NF-κB inflammatory cascade, and restored occludin and keratin levels (*p* < 0.01 vs. the model group). It also exerted anti-*Microsporum gypseum* activity, with a minimum inhibitory concentration (MIC) of 92.36 ± 1.03 μg/mL, which alleviated inflammation and pruritus associated with dermatomycosis. In barrier-damaged mice, topical application of **AKE** recovered 81.2% of epidermal integrity (*p* < 0.001 vs. the model group) and normalized skin biomarkers. UHPLC-ESI-HRMS/MS combined with molecular docking identified atractylenolide I-II as potential bioactive compounds. In summary, **AKE** repairs damaged epidermis through multi-target modulation, indicating promising potential as a natural functional candidate for skin barrier repair.

## 1. Introduction

The skin, which is a significant and intricate organ, functions mainly as the primary defense barrier between the organism and its external surroundings. It defends the body against a broad spectrum of pathogenic threats, such as toxic chemicals, UV radiation, mechanical damage, allergens, and pathogens [1]. The epidermal barrier plays a crucial role in protection by maintaining skin functional homeostasis. This skin barrier comprises unique anatomical layers: the stratum basale, stratum spinosum, stratum granulosum, and stratum corneum [2,3]. An impaired skin barrier results in higher transepidermal water loss (TEWL), exposing the skin to greater risk of external damage. This triggers symptoms frequently linked to sensitive skin, including dryness, wrinkles, redness, burning sensations, and swelling [4]. Epidemiological studies reveal that sensitive skin is linked to skin barrier dysfunction. Globally, 71% of adults report sensitive skin of any severity, while 40% experience moderate to severe symptoms. This condition significantly compromises quality of life and drives substantial demand for mild skin barrier repair ingredients [5,6]. Without timely intervention, persistent disruption of barrier function may further progress to severe dermatological problems, significantly impacting people’s quality of life [7].

Since 1949, synthetic therapeutic agents, notably hydrocortisone and tretinoin, have been extensively utilized to manage inflammatory dermatoses such as atopic dermatitis and psoriasis [8]. These agents are widely utilized and exhibit high clinical efficacy [9,10]. However, their application is limited in people with sensitive skin due to their side effects, such as causing burning, redness, swelling, and itching [7]. Alternatively, bioactive compounds obtained from medicinal and edible herbs are recognized for their safety, gentleness, and minimal irritation, making them attractive options for treating skin barrier damage.

**Figure 1:**
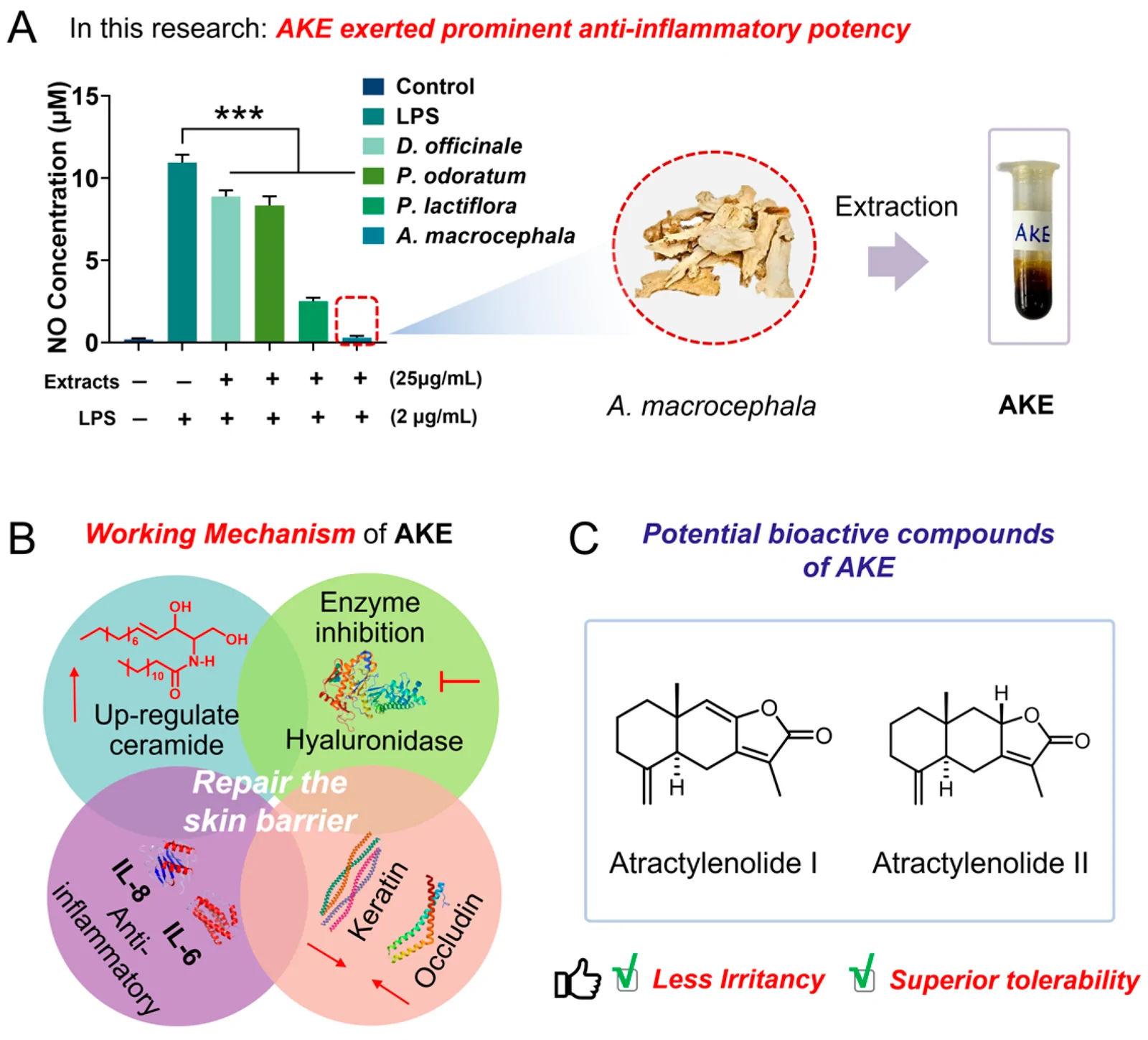
(**A**) Inhibitory effects of four herbal extracts on LPS-induced NO production; (**B**) schematic illustration of the multi-target mechanism of **AKE** in skin barrier repair; (**C**) chemical structures of potential bioactive compounds of **AKE** and its advantages. *** *p* < 0.001, compared with the model group. Abbreviations: **AKE**, *Atractylodes macrocephala* Koidz extract; NO, nitric oxide; LPS, lipopolysaccharide; *D. officinale*, *Dendrobium officinale Kimura* et Migo; *P. odoratum*, *Polygonatum odoratum* (Mill.) Druce; *P. lactiflora*, *Paeonia lactiflora* Pall; *A. macrocephala*, *Atractylodes macrocephala* Koidz.

Traditional Chinese medicine has documented numerous herbal candidates with skin protective properties, but systematic pharmacological validation remains limited, especially regarding multi-target molecular mechanisms underlying barrier restoration. In the present work, four medicinal and edible herbs were selected as candidate materials for repairing skin barriers, including *Atractylodes macrocephala* Koidz. (*A. macrocephala*), *Paeonia lactiflora* Pall. (*P. lactiflora*), *Dendrobium officinale Kimura* et Migo (*D. officinale*), and *Polygonatum odoratum* (Mill.) Druce (*P. odoratum*). Their effects and fundamental mechanism in repairing the epidermal barrier and alleviating sensitive skin symptoms are still largely uninvestigated. Preliminary nitric oxide (NO) inhibitory assays demonstrated that that extracts from *A. macrocephala* (**AKE**) and *P. lactiflora* (**PPE**) exerted prominent anti-inflammatory potency (Figure 1A) [11]. In this study, *P. lactiflora* extract (**PPE**) was included only as a comparative extract for preliminary comparison and partial mechanistic study, while *A. macrocephala* extract (**AKE**) was set as the primary focus of this study. *A. macrocephala* is a well-known medicinal herb. Current research predominantly focuses on its gastrointestinal regulatory and immunomodulatory properties, while there is still limited comprehensive mechanistic study on its role in repairing the epidermal barrier. In order to address this research gap, we systematically evaluated the skin repair efficacy of **AKE** using multiple research strategies, including in vitro biochemical assays, keratinocyte injured models, spectroscopic interaction analysis, molecular docking simulations, and the tape stripping-induced mouse model of skin barrier impairment. Mechanistically, this study aimed to elucidate the multi-target regulatory mechanisms of **AKE** on damaged epidermal barrier. Our findings demonstrated that **AKE** could promote ceramide biosynthesis, inhibit hyaluronidase (HAase), collagenase and elastase activities, stabilize the level of tight junction proteins, suppress NF-κB-mediated inflammatory responses, and exhibit anti-dermatophyte bioactivity (Figure 1B). UHPLC-ESI-HRMS/MS analysis and molecular docking simulation were further implemented to identify the potential bioactive constituents in **AKE** (Figure 1C). Collectively, this study demonstrated that **AKE** significantly restored damaged epidermal integrity, highlighting its potential as a functional ingredient for skin barrier repair. This study systematically elucidated the multi-target mechanism of **AKE** in skin barrier repair, filling the relevant research gap and establishing a theoretical foundation for the future development and application of **AKE**.

## 2. Results and Discussion

### 2.1. Medicinal Plant Selection and Extraction

Excessive production of NO is known to cause pronounced inflammatory response, given its function as an essential physiological signaling intermediate. The pharmacological suppression of NO synthesis offers a plausible avenue for dampening inflammatory responses [11]. Thus, we evaluated a panel comprising *P. odoratum*, *A. macrocephala*, *D. officinale*, and *P. lactiflora* for their ability to inhibit NO generation in RAW264.7 macrophages stimulated with lipopolysaccharide. The results revealed a marked disparity in the suppressive efficacies of these extracts. Among the tested candidates, *A. macrocephala* and *P. lactiflora* emerged as the most potent inhibitors, achieving respective suppression rates of 97.39% and 77.06% (Figure 1A). These results demonstrated that these extracts had considerable anti-inflammatory potential. Therefore, the present study mainly focused on **AKE** extract to further investigate its potential efficacy for skin barrier restoration. **PPE** extract was used as a control extract for preliminary comparison and partial mechanistic exploration.

### 2.2. Cell Viability Assay

Keratinocytes constitute the major component of the epidermal cell population and are indispensable for maintaining the cutaneous permeability barrier [12]. We first evaluated the cytotoxicity of extracts against HaCaT cells in order to assess the biocompatibility. HaCaT cells were treated with graded concentrations of extract, spanning 0.5, 2.5, 12.5, and 62.5 μg/mL. As depicted in Figure S1, neither extract produced appreciable adverse effects over the examined range. More importantly, at levels not exceeding 12.5 μg/mL, both extracts markedly stimulated the proliferation of HaCaT cells (*p* < 0.05). Taken together, these results revealed that extracts possessed favorable biocompatibility, satisfying the fundamental prerequisite for subsequent development and application research.

### 2.3. Wound-Healing Activity

**Figure 2:**
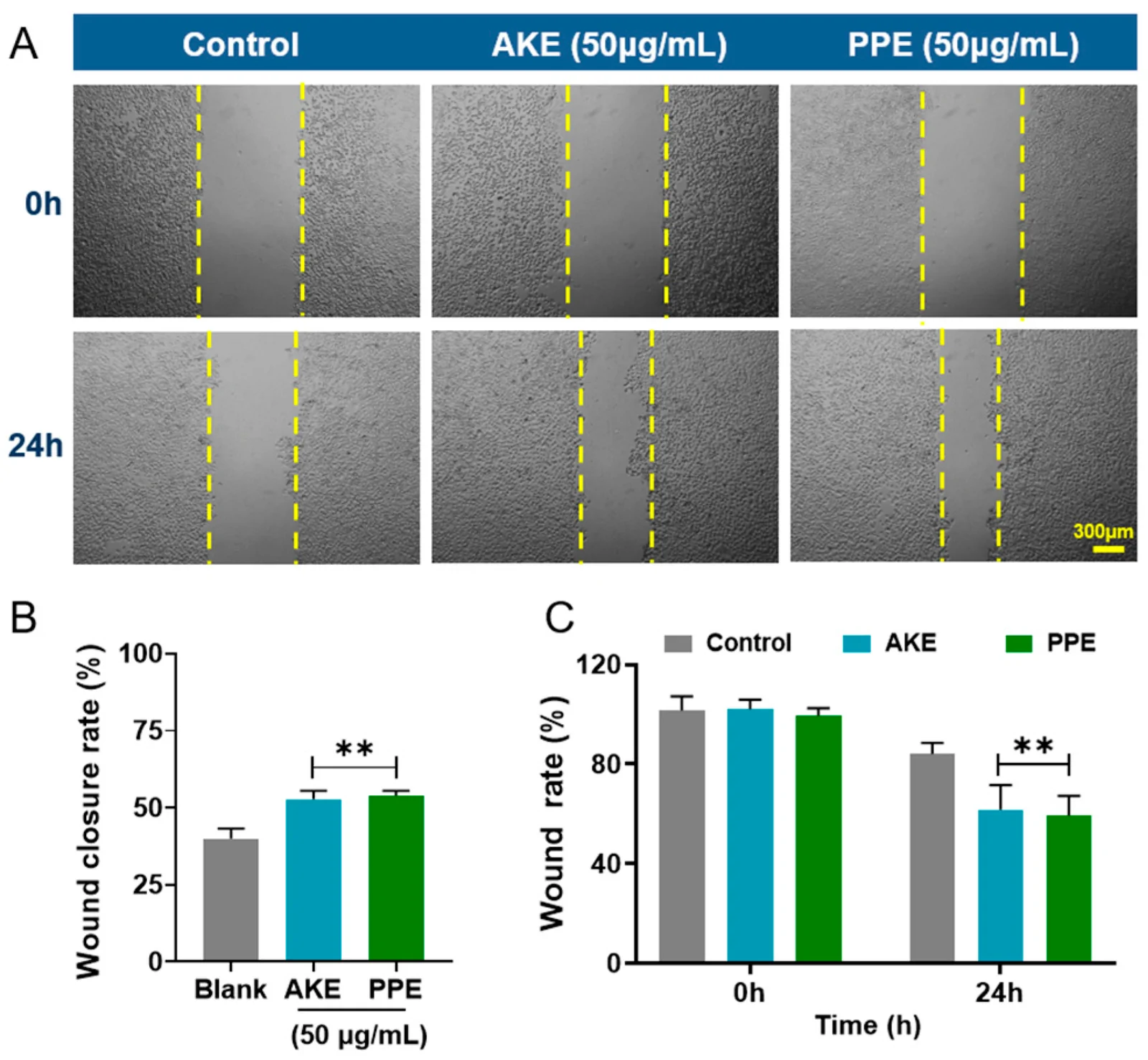
Evaluation of the wound-healing effect of the extracts on HaCaT cells. (**A**) Representative bright field scratch wound micrographs at 0 h and 24 h post-scratching; yellow dashed lines mark wound boundaries. (**B**) Quantified wound closure rate (%) after 24 h incubation. (**C**) Time-dependent relative wound area percentage at 0 h and 24 h. Data are expressed as a comparison, as a percentage of wound closure relative to control, between the wound area at 0 and 24 h. ** *p* < 0.01, compared to control. Magnification = 10×; scale bar = 300 µm. Abbreviations: **AKE**, *Atractylodes macrocephala* Koidz extract; **PPE**, *Paeonia lactiflora* Pall. extract.

When the skin barrier is damaged, epidermal keratinocytes secrete cytokines that stimulate endothelial cell migration within wound sites. Accordingly, cell migration serves as an indispensable component of wound healing cascades [13]. The scratch wound healing assay was performed to evaluate the migratory capacity of HaCaT cells following treatment with the tested extracts. This assay evaluated cell migration and wound closure efficiency by detecting the repopulation of artificially induced cell-free gaps within confluent cell monolayers [14]. As shown in Figure 2, the untreated control group showed a lower gap closure rate compared to the extract-treated groups. After 24 h of exposure to the 50 µg/mL of **AKE** and **PPE**, increased wound healing was observed. Both extracts promoted cell migration, with the migration rate increased by over 10% relative to the untreated control group (*p* < 0.01). At present, many studies have reported the wound-healing efficacy of plant-derived bioactive extracts. For example, the extract of *Lotus rectus* L. achieved more than 20% improvement in scratch closure at concentrations of 100–200 μg/mL following 18 h of incubation [15]. Moreover, extracts from *Galenia africana* and *Tulbaghia violacea* exerted strong migration-promoting effects in HaCaT keratinocytes at 40 μg/mL and 60 μg/mL, respectively. In comparison, **AKE** displayed prominent promigratory activity at a lower effective concentration of 50 μg/mL [16]. Collectively, our study indicated that **AKE** could effectively facilitate in vitro wound closure in HaCaT cells, indicating that **AKE** supported the process that is essential for skin regeneration.

### 2.4. Effects of Extracts on ROS Secretion in SLS-Induced HaCaT Cells

**Figure 3:**
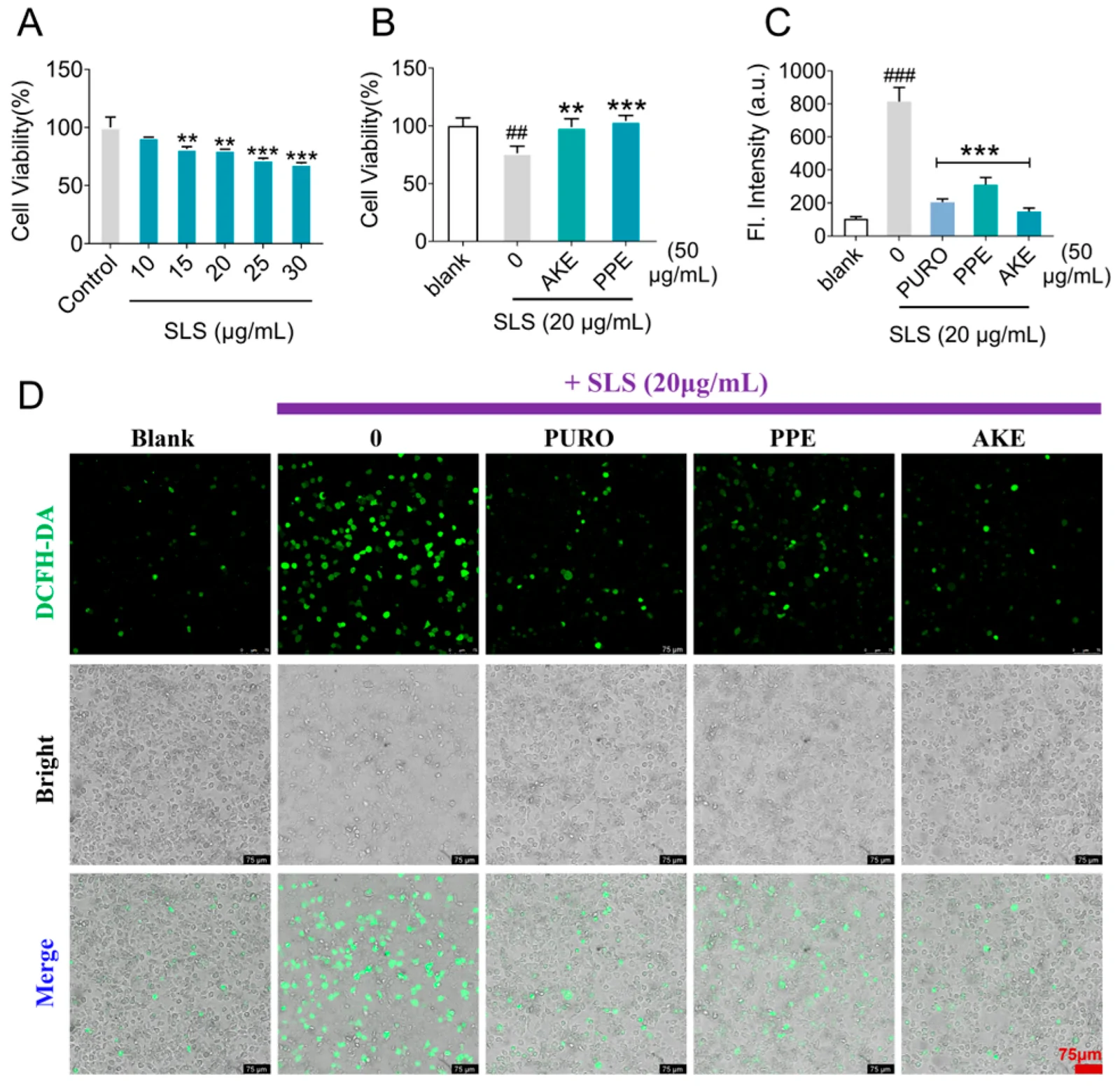
(**A**) Cell viability of HaCaT cells treated with SLS. (**B**) Extracts increased the cell viability in SLS-treated HaCaT cells. (**C**) Quantification of fluorescence intensity. (**D**) Images of DCFH-DA fluorescence observed via Confocal Microscopy, scale bar = 75 µm. Compared with the control group: ** *p* < 0.01 and *** *p* < 0.001. Compared with the blank group: ^##^ *p* < 0.01 and ^###^ *p* < 0.001. Abbreviations: **AKE**, *Atractylodes macrocephala* Koidz extract; **PPE**, *Paeonia lactiflora* Pall. extract; PURO, *Prinsepia utilis* Royle oil; SLS, sodium lauryl sulfate; DCFH-DA, 2′,7′-Dichlorodihydrofluorescein diacetate.

Sodium lauryl sulfate (SLS), an irritant surfactant for skin, is capable of inducing epidermal injury and allergic sensitization [17]. Mechanistically, SLS can disrupt the structure of intercellular tight junctions, damage the epidermal barrier and subsequently provoke oxidative stress and inflammatory responses in keratinocytes. We constructed the damaged model by exposing HaCaT cells to rising SLS concentrations. As illustrated in Figure 3A, there was a progressive decline with increasing SLS concentrations. Notably, 20 μg/mL SLS induced significant cytotoxicity and was therefore selected as the injury condition for subsequent related assays. Cellular viability dropped to 76.24% following SLS stimulation, while supplementation with the tested extracts markedly reduced cytotoxic damage stimulated by SLS (Figure 3B). DCFH-DA staining was then adopted to assess intracellular ROS accumulation. The quantitative fluorescence intensity results (Figure 3C) and confocal micrograph images (Figure 3D) illustrated that SLS exposure induced a substantial reactive oxygen species (ROS) burst in the model group. After intervention with extracts, intracellular ROS were efficiently suppressed. Among all tested groups, **AKE** exhibited reliable ROS scavenging capacity against oxidative stress in damaged keratinocytes. Accordingly, the antioxidant activity of **AKE** was regarded as one primary mechanism for epidermal barrier restoration. Consistent with our present findings, Gardeniae Fructus extract was previously reported to alleviate SLS-triggered intracellular ROS accumulation in HaCaT keratinocytes under 15 μg/mL SLS injury conditions [17]. Notably, our study adopted a more severe damage model with 20 μg/mL SLS stimulation. Even under this stronger oxidative insult, **AKE** achieved efficient suppression of intracellular ROS burst, demonstrating its competitive antioxidative potency. These comparative results further supported that **AKE** could effectively inhibit excessive ROS provoked by surfactant-mediated epidermal injury, thereby relieving oxidative-driven keratinocyte damage and establishing the foundation for the subsequent restoration of barrier functions.

### 2.5. Evaluation of the Promoting Effect on Ceramide Synthesis

**Figure 4:**
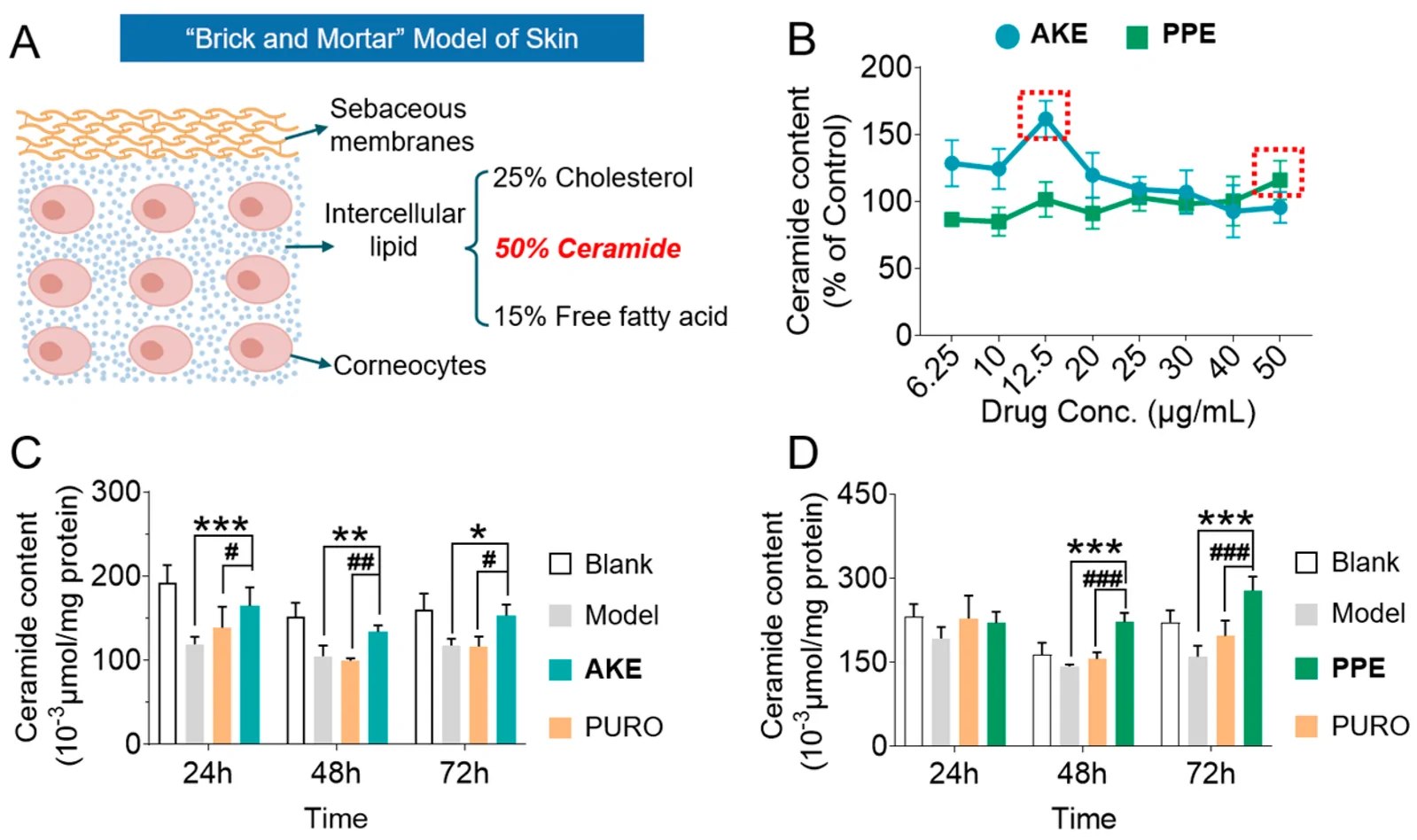
(**A**) Schematic representation of the “Brick and Mortar” model of the skin epidermal barrier. (**B**) Effect of extracts at different concentrations on ceramide levels in HaCaT cells; The red dashed box indicates the optimal working concentration of the extracts. (**C**,**D**) Time-dependent effects of **AKE** (12.5 μg/mL) and **PPE** (50.0 μg/mL) on ceramide content in HaCaT cells at 24, 48, and 72 h. *, ^#^ *p*< 0.05, **, ^##^ *p*< 0.01, ***, ^###^ *p*< 0.001. Abbreviations: **AKE**, *Atractylodes macrocephala* Koidz extract; **PPE**, *Paeonia lactiflora* Pall. ex-tract; PURO, *Prinsepia utilis* Royle oil.

Based on the classic “brick and mortar” model of the skin epidermal barrier, the epidermal stratum corneum is visualized as an array of corneocytes (the “bricks”) interspersed within a continuous lipid-rich intercellular compartment a (the “mortar”), which is largely comprised of ceramides (~50%), cholesterol (~25%), and free fatty acids (~15%) (Figure 4A). Ceramides, one of the glycolipids, perform a dual function: maintaining the structural rigidity of the membrane, while acting as a secondary messenger to regulate cellular renewal and skin homeostasis [3]. Extensive research has consistently showed that the deficiency in ceramide levels will induce increased TEWL, which can lead to impaired barrier function and be accompanied by symptoms such as dryness, aging, itching [18].

To evaluate the modulatory effects of extracts in ceramide biosynthesis, HaCaT cells were incubated with varying dosages for each extract. The curves illustrated in Figure 4B revealed different regulatory capabilities between the two extracts. Treatment with **AKE** consistently increased ceramide production at the tested concentration range of 6.25 to 40 μg/mL, demonstrating markedly better efficacy compared with the commercial reference *Prinsepia utilis* Royle oil (PURO), even surpassing PURO at matched incubation time points. Specifically, the strongest response to **AKE** was observed at 12.5 μg/mL, resulting in a 1.61-fold increase relative to the baseline control group. By contrast, **PPE** exhibited relatively weaker protective effects, reaching its peak activity of merely a 1.15-fold elevation at 50 μg/mL. Based on these results, 12.5 μg/mL **AKE** and 50 μg/mL **PPE** were selected as suitable working concentrations for subsequent research. Moreover, we quantified intracellular ceramide levels at 24, 48, and 72 h treatment using the optimal concentration determined for each extract. As shown in Figure 4C,D, ceramide levels were markedly downregulated in the SLS damaged model group, confirming the disruption of sphingolipid metabolism and depletion of endogenous ceramide. After 24 h intervention, **AKE** significantly restored ceramide concentration, and its effect outperformed the PURO positive control group (*p* < 0.05). Notably, even during 48 or 72 h of incubation, **AKE** sustained a prominent upregulation of ceramides, exhibiting the strongest activity among the three intervention groups. Conversely, **PPE** displayed delayed enhanced activity, with no significant ceramide elevation tested at 24 h. This result indicated the promising advantages of **AKE** as candidate modulators targeting ceramide-related signaling pathways, suggesting its great potential to preserve epidermal barrier integrity and skin homeostasis.

Compared with previously reported plant-derived agents that modulate ceramide metabolism, *Gentiana lutea* extract elevated only the levels of glucosylceramide and ceramide AS/AdS subclass, with no change in total cellular ceramide abundance [19]. The natural modulator β-sitosterol 3-O-D-glucoside increased total ceramide predominantly through selective accumulation of the acyl-ceramide Cer [EOS], with limited regulatory effects on other ceramide subspecies [20]. In contrast, **AKE** exerted superior regulatory performance. **AKE** stimulated ceramide production in a dose-dependent manner over a wide concentration range. It rapidly reversed intracellular ceramide depletion resulting from SLS-mediated barrier injury as early as 24 h, with its upregulating activity persisting for as long as 72 h. Such properties of rapid and sustaining restoration of total ceramide content over the time-course confer upon **AKE** distinct advantages in epidermal barrier recovery compared with previously reported botanical candidates.

### 2.6. Inhibition of Enzyme Activity In Vitro

#### 2.6.1. Hyaluronidase Inhibitory Activity

**Figure 5:**
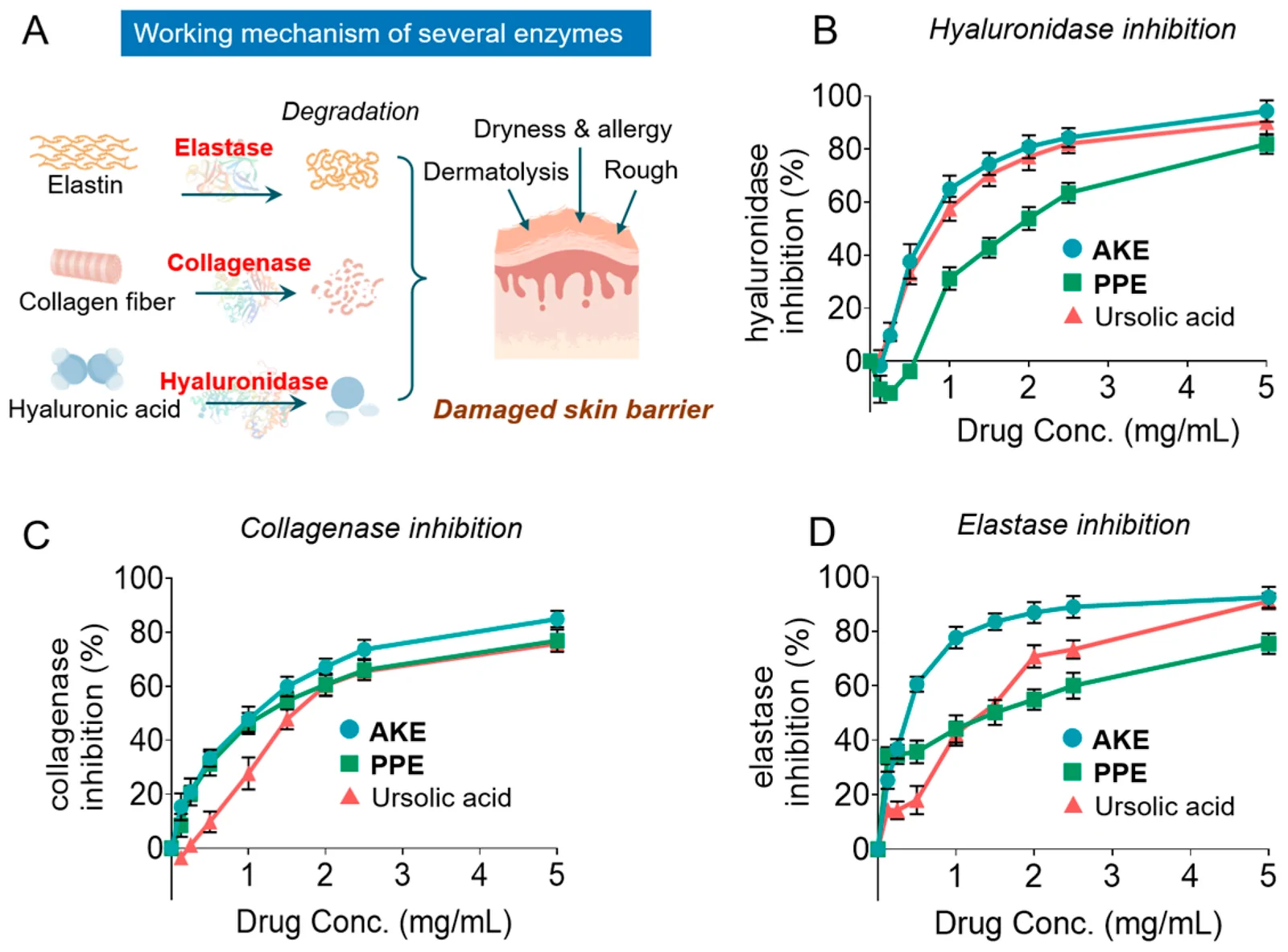
(**A**) Mechanisms of HAase, elastase, and collagenase in skin barrier degradation. (**B**–**D**): Inhibitory activity of the extracts on HAase (**B**), collagenase (**C**), and elastase (**D**) enzymes. Abbreviations: **AKE**, *Atractylodes macrocephala* Koidz extract; **PPE**, *Paeonia lactiflora* Pall. extract; PURO, *Prinsepia utilis* Royle oil; HAase, hyaluronidase.

As a vital extracellular matrix (ECM) enzyme ubiquitous in human tissues, HAase catalyzes hyaluronic acid degradation, which triggers dermal irritation, allergic symptoms and skin aging (Figure 5A) [21]. Thus, effective inhibition of this enzyme is indispensable for maintaining cutaneous homeostasis. Given the drawbacks of synthetic inhibitory agents, natural plant-based inhibitors are attracting growing research interest for pharmaceutical development and skincare formulations. To assess the HAase inhibitory capacity of extracts, biochemical enzyme inhibition assays were performed at concentrations ranging from 0.125 to 5.0 mg/mL. As shown in Figure 5B, extracts suppressed HAase activity dose-dependently, with the inhibitory rate close to 80%. **AKE** exhibited prominent HAase-suppressing capacity, with an IC_50_ value of 0.5544 mg/mL, a better inhibitory performance than the positive control ursolic acid (IC_50_ = 0.6975 mg/mL). By comparison, **PPE** exerted relatively weak HAase inhibition, with an IC_50_ value of 1.293 mg/mL (Figure S2). These findings indicated that **AKE** can serve as an excellent natural inhibitor to alleviate inflammatory reactions, allergic symptoms, skin dryness and skin aging.

#### 2.6.2. Collagenase Inhibitory Activity

Collagen, the predominant structural protein of human skin, serves as the primary structural protein responsible for skin firmness, hydration and youthful morphology. Collagenase, an endogenous cutaneous enzyme, catalyzes collagen breakdown. Abnormally high levels of collagenase activity can degrade connective tissue proteins and impair dermal structure, thereby accelerating the formation of wrinkles, stimulating inflammatory responses, and inhibiting wound healing (Figure 5A) [22,23]. Accordingly, developing natural collagenase inhibitors is essential for skin anti-aging research. In this study, we evaluated the inhibitory effects of extracts against collagenase through biochemical enzyme activity inhibition assays. As shown in Figure 5C, extracts suppressed collagenase activity in a dose-dependent manner. Among them, **AKE** achieved significant inhibitory potency, with an IC_50_ value of 1.220 mg/mL, superior to ursolic acid (1.228 mg/mL; Figure S2). At concentrations ranging from 2.00 to 5.00 mg/mL, **AKE** also maintained excellent performance. Collectively, these results confirmed **AKE** as a powerful natural collagenase inhibitor, with great prospects for preserving skin structural stability and hydration.

#### 2.6.3. Elastase Inhibitory Activity

Elastase belongs to the serine protease family, responsible for hydrolyzing elastin and multiple extracellular matrix components. Abnormally elevated elastase activity reduces skin elasticity, induces transepidermal water loss, and impairs epidermal structural integrity (Figure 5A) [22,23]. To evaluate the efficacy of extracts in inhibiting wrinkle formation, biochemical assays were performed to determine their elastase inhibitory activity. As displayed in Figure 5D, extracts suppressed elastase activity in a dose-dependent manner. Remarkably, **AKE** possessed superior inhibitory potency with IC_50_ of 0.4304 mg/mL, which was markedly superior to those of ursolic acid (IC_50_ = 1.434 mg/mL) and **PPE** (IC_50_ = 2.839 mg/mL). These results highlighted **AKE** as a powerful natural elastase inhibitor, which can protect elastin from degradation, alleviate skin dryness and wrinkles.

Notably, compared with the recently reported extract of red clover, which exhibited an IC_50_ value of 22.443 mg/mL against HAase and 0.602 mg/mL for elastase, **AKE** displayed substantially superior inhibitory potency against these two extracellular matrix-degrading enzymes [24]. Specifically, **AKE** possessed a lower IC_50_ toward HAase (0.5544 mg/mL) and also outperformed red clover extract in elastase inhibition (0.4304 mg/mL). Furthermore, the reported studies have revealed that red clover extract failed to inhibit collagenase, whereas **AKE** in this study exerted potent collagenase inhibitory activity with an IC_50_ of 1.220 mg/mL. Collectively, these comparative results further highlighted the strong competitive advantages of **AKE** as a novel multi-target natural skin barrier repair agent.

### 2.7. Anti-Inflammatory Activity

**Figure 6:**
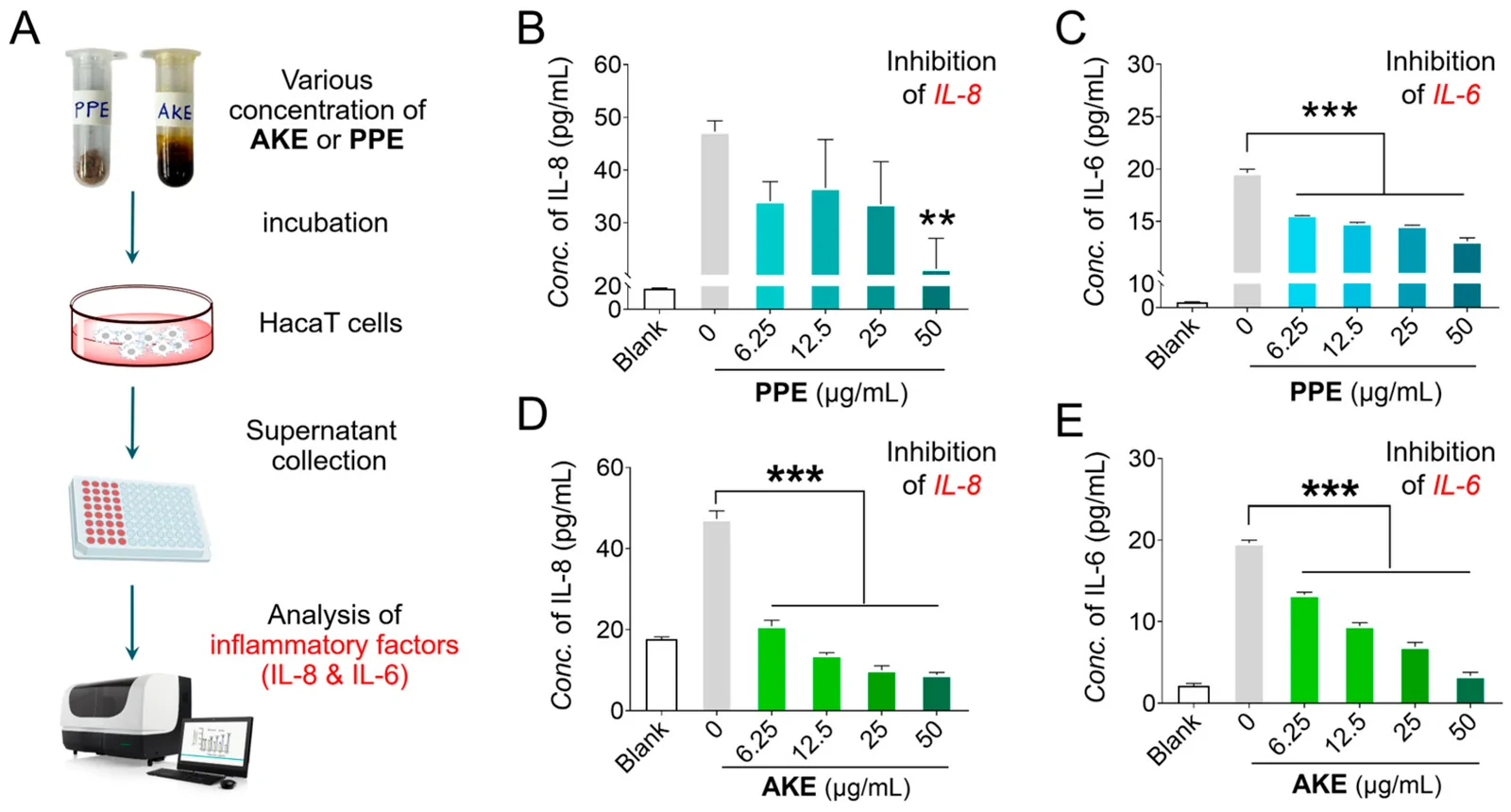
(**A**) Schematic of the experimental workflow for the analysis of inflammatory factors. (**B**–**E**) Inhibitory effects of the extracts at various concentrations on IL-6 and IL-8 production. ** *p* < 0.01, *** *p* < 0.001, compared with the model group. Abbreviations: **AKE**, *Atractylodes macrocephala* Koidz extract; **PPE**, *Paeonia lactiflora* Pall. ex-tract; PURO, *Prinsepia utilis* Royle oil.

Ceramides, critical constituents of the epidermal lipid matrix, serve as secondary messengers that regulate keratinocyte proliferation, differentiation and inflammation. When the metabolism of ceramides is disrupted, it can induce inflammatory skin disorders [3]. As vital inflammatory mediators, IL-6 and IL-8 participate in various of skin inflammatory responses. IL-6 participates in the initiation of local inflammatory cascades, whereas IL-8 serves as a potent chemokine that aggravates cellular stress injury [25]. Suppressing the excessive secretion of these two cytokines is a critical indicator for evaluating the cytoprotective and anti-inflammatory capacities of extracts. We measured IL-6 and IL-8 production in HaCaT cells exposed to extracts at 6.25–50 μg/mL for 24 h to characterize their anti-inflammatory effects (Figure 6B–E). In this study, IL-6 and IL-8 concentrations were markedly elevated in the SLS damage model group, confirming that the damaging stimulus had successfully triggered an intracellular inflammatory response. As shown in Figure 6B–E, extracts showed concentration-dependent inhibitory activity. **AKE** exhibited significant suppression of IL-6 and IL-8 production at 6.25 μg/mL. The inhibitory effects were further enhanced as concentration increased, reaching maximum inhibition at 50 μg/mL. The levels of inflammatory factors in the **AKE** treatment group were significantly lower than those in the injury model control group (*p* < 0.001). By comparison, **PPE** required higher working concentration to achieve potent inhibitory activity against inflammatory cytokines. Combined with our previous studies on ceramide quantification, these findings demonstrated that **AKE** possessed more potent anti-inflammatory capacity, supporting its development as a promising natural active ingredient targeted at skin barrier repair. Furthermore, the dose–response characteristics of **AKE** and **PPE** implied a tight correlation between ceramide metabolism and its anti-inflammatory properties.

In comparison with reported botanical ingredients that target ceramide metabolism, *Gentiana lutea* extract failed to elevate total cellular ceramide content and partly rescued expression of elongases (ELOVL4) with borderline statistical significance (*p* = 0.07), and there was no direct evidence for its inhibitory effect on keratinocyte-derived IL-6 and IL-8 [19]. Distinct from such agents, **AKE** exerted dual beneficial bioactivities of reversing SLS-provoked ceramide depletion and simultaneously inhibited excessive IL-6 and IL-8 production even at relatively low concentrations, which highlighting its advantage as a multi-functional candidate for barrier-damaged skin.

### 2.8. Regulatory Effect on the Level of Tight Junction Proteins

**Figure 7:**
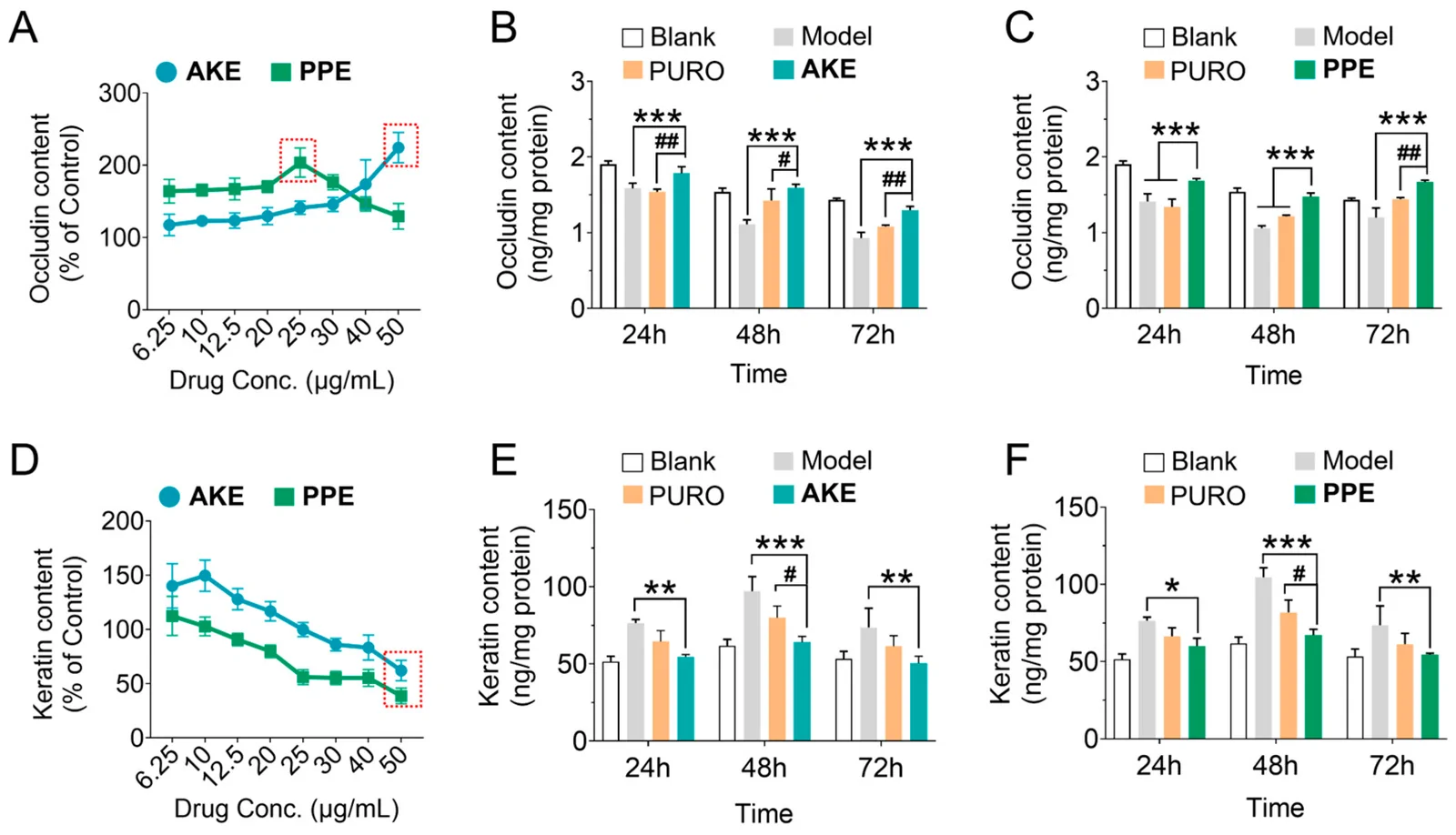
(**A**) Effect of the extracts at various concentrations on occludin content in HaCaT cells (24 h). (**B**,**C**) Time-dependent effects of **AKE** (50.0 μg/mL) and **PPE** (25.0 μg/mL) on occludin content in HaCaT cells at 24/48/72 h. (**D**) Effect of the extracts at various concentrations on keratin content in HaCaT cells (24 h). (**E**,**F**) Time-dependent effects of **AKE** (50.0 μg/mL) and **PPE** (50.0 μg/mL) on keratin content in HaCaT cells at 24/48/72 h. The red dashed box represents the optimal working concentration of the extract. * *p* < 0.05, ** *p* < 0.01, and *** *p* < 0.001, compared with the model group; ^#^ *p* < 0.05, ^##^ *p* < 0.01, compared with the positive control group. Abbreviations: **AKE**, *Atractylodes macrocephala* Koidz extract; **PPE**, *Paeonia lactiflora* Pall. extract; PURO, *Prinsepia utilis* Royle oil.

Tight junction (TJ) proteins are vital for sustaining epidermal barrier homeostasis and responsible for protecting the stratum corneum from damage [2]. As an indispensable TJ protein component, occludin regulates the formation of functional skin barriers. When the skin barrier is damaged, occludin levels are drastically downregulated or undetectable in the stratum corneum [26,27,28]. Keratins function as core fibrous structural elements maintaining epidermal integrity, hair and nail tissues. Excessive keratin expression may lead to the occurrence of keratinization-related skin disorders [29,30]. To investigate the regulatory mechanism of the extracts on TJ proteins, we quantified occludin and keratin levels in HaCaT keratinocytes by ELISA assay. The results are shown in Figure 7A–F. SLS stimulation decreased occludin levels and induced excessive keratin synthesis, which verified the disruption of barrier homeostasis. As shown in Figure 7A–F, extracts elevated occludin secretion and restrained abnormal keratin synthesis across 6.25–50 μg/mL. With respect to occludin regulation, **AKE** exerted favorable regulatory efficacy, with occludin expression increasing 2.24-fold relative to the untreated group upon **AKE** treatment at 50 μg/mL. Within 72 h, the upregulatory effect of **AKE** on occludin was even significantly stronger than that of the control group PURO (*p* < 0.05). In contrast, **PPE** only reached a maximum 2.04-fold induction at the optimal concentration and only outperformed PURO in terms of regulatory effects at 72 h (*p* < 0.05) (Figure 7A–C). With respect to keratin regulation, extracts markedly downregulated keratin overexpression at 50 μg/mL (Figure 7D). At this concentration, their regulatory effects differed significantly from those of the untreated control at 24, 48, and 72 h post-treatment (*p* < 0.05) (Figure 7E,F).

Notably, previous studies had reported that *Prinsepia utilis* Royle polysaccharide could restore the expression of disrupted TJ proteins in SLS-damaged skin models [31]. Nevertheless, the study lacked quantitative fold-change results and serial time process evaluation at the cellular level. In the present system, **AKE** not only dose-dependently elevated occludin abundance, but also persistently restrained SLS-induced excessive keratin synthesis over 24 h to 72 h. Furthermore, **AKE** produced a higher fold induction of occludin at 50 μg/mL and exhibited statistically stronger efficacy than PURO at 72 h, highlighting its unique capacity to simultaneously enhance the synthesis of occludin and reduce the excessive accumulation of abnormal keratin under barrier-damaged conditions. Moreover, the regulatory effect of **AKE** was significantly superior to PURO (*p* < 0.01). These findings demonstrated that **AKE** could restore skin barrier function and reduce epidermal hyperplasia by enhancing the expression of occludin and suppressing the overexpression of abnormal keratins. **AKE** showed great potential as an effective ingredient for reconstructing damaged epidermal barriers.

### 2.9. Effects of Extracts on Epidermal Barrier Biomarkers and NF-κB Inflammatory Pathway

**Figure 8:**
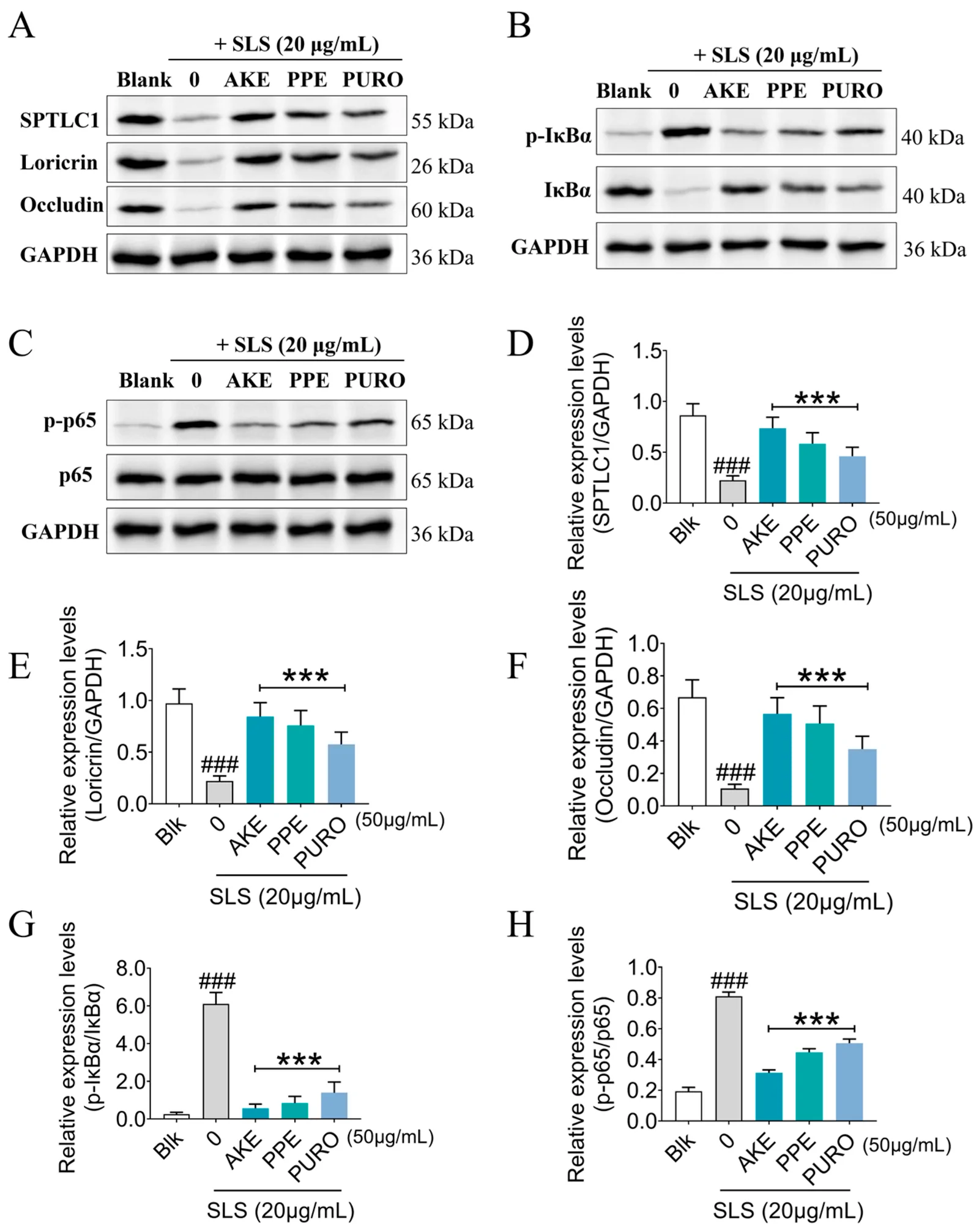
(**A**–**C**) Representative Western blot images of the regulatory effects of extracts on the expression of SPTLC1, loricrin, occludin, p-IκBα/IκBα, and p-p65/p65 signaling pathway-related proteins. (**D**–**H**) Quantification of relative protein expression. HaCaT cells were separated into the blank control group (Blank), SLS-induced barrier injury model group, **AKE**-treated group, **PPE**-treated group and positive control PURO group. All injury groups were stimulated with 20 μg/mL SLS. *** *p* < 0.001, compared with the model group; ^###^ *p* < 0.001 compared with the blank control group. Abbreviations: SLS, sodium lauryl sulfate; **AKE**, *Atractylodes macrocephala* Koidz extract; **PPE**, *Paeonia lactiflora* Pall. extract; PURO, *Prinsepia utilis* Royle oil; SPTLC1, serine palmitoyltransferase long chain-1.

Prior to this study, we examined the secretion of barrier proteins and inflammatory cytokines, lacking direct validation of regulatory enzymes and inflammatory signaling pathways at the protein level. To further investigate the molecular mechanism of extract-relieved epidermal barrier damage, Western blot analysis was performed to quantify the expression levels of related proteins. Representative Western blot images and quantitative grey scale analysis are displayed in Figure 8. The integrity of epidermal barriers depends on functional tight junction components and ceramide synthesis-related proteins. Specifically, serine palmitoyltransferase long chain-1 (SPTLC1) participates in ceramide biosynthesis, while loricrin constitutes the principal skin structural protein and occludin maintains tight junction stability [17,32]. Compared with the blank control group, stimulation with 20 μg/mL SLS markedly downregulated the protein levels of SPTLC1, loricrin and occludin (*p* < 0.001). The significant reduction in these proteins indicated severe destruction of epidermal tight junctions, confirming the successful construction of the keratinocyte epidermal barrier injury model. Intervention with extracts markedly reversed the SLS-induced aberration of these biomarker proteins (*p* < 0.001), with **AKE** exhibiting the most prominent regulatory effect among all treatment groups. As a critical enzyme for epidermal ceramide synthesis, SPTLC1 was sharply depleted in the model group, which explained the decreased epidermal ceramide levels observed above. Extract intervention effectively restored SPTLC1 protein expression to a normal level, demonstrating that **AKE** could improve epidermal lipid barrier function by recovering SPTLC1-mediated ceramide biosynthesis. In addition, loricrin and occludin, important biomarkers of epidermal barrier integrity, were significantly suppressed in the damaged model group. **AKE** intervention effectively upregulated the expression of these two proteins, further promoting the repair of impaired epidermal barrier structure and function. These findings indicated that **AKE** exerted a protective effect against SLS-induced epidermal barrier dysfunction by restoring the expression of core barrier functional proteins.

The NF-κB signaling pathway, a critical regulatory axis linking epidermal barrier disruption and cutaneous inflammatory activation, regulates the secretion of proinflammatory cytokines IL-6 and IL-8 in damaged skin [33]. Western blot results revealed that the phosphorylation ratios of p-IκBα/IκBα and p-p65/p65 were sharply elevated after SLS intervention (*p* < 0.001) (Figure 8). SLS-mediated IκBα phosphorylation accelerated its intracellular degradation, releasing the p65 subunit and facilitating p65 phosphorylation. This cascade indicated robust activation of the NF-κB signaling pathway and the subsequent initiation of inflammatory responses. Relative to the SLS-damaged group, extracts dramatically decreased the phosphorylation levels of IκBα and p65 (*p* < 0.001), with **AKE** exerting the strongest regulatory effect among all intervention groups. These results indicated that **AKE** exerted its anti-inflammatory effects by inhibiting the phosphorylation of p65 and the activation of the NF-κB signaling pathway. This conclusion further elucidated the molecular mechanism behind the preceding inflammatory factor assays, in which **AKE** inhibited the phosphorylation of p-IκBα/IκBα and p-p65/p65, thereby reducing the levels of downstream inflammatory cytokines IL-6 and IL-8.

Collectively, these findings demonstrated that **AKE** could relieve SLS-provoked barrier damage, complementing our previous phenotypic and cytokine-related results. In contrast to most plant-derived skincare ingredients, which generally function through a single pathway, **AKE** displayed unique multi-target efficacy in the present study. Its protective mechanism involved restoring tight junction proteins and ceramide synthesis-associated proteins, while simultaneously interrupting the self-perpetuating cycle between epidermal barrier impairment and sustained inflammatory responses through inhibition of NF-κB pathway activation. This multi-target protein regulatory network systematically revealed the biological function of **AKE** in maintaining epidermal homeostasis, and substantiated its potential as a promising natural derived candidate material for the prevention of skin irritant injury.

### 2.10. Fluorescence Quenching Measurements

**Figure 9:**
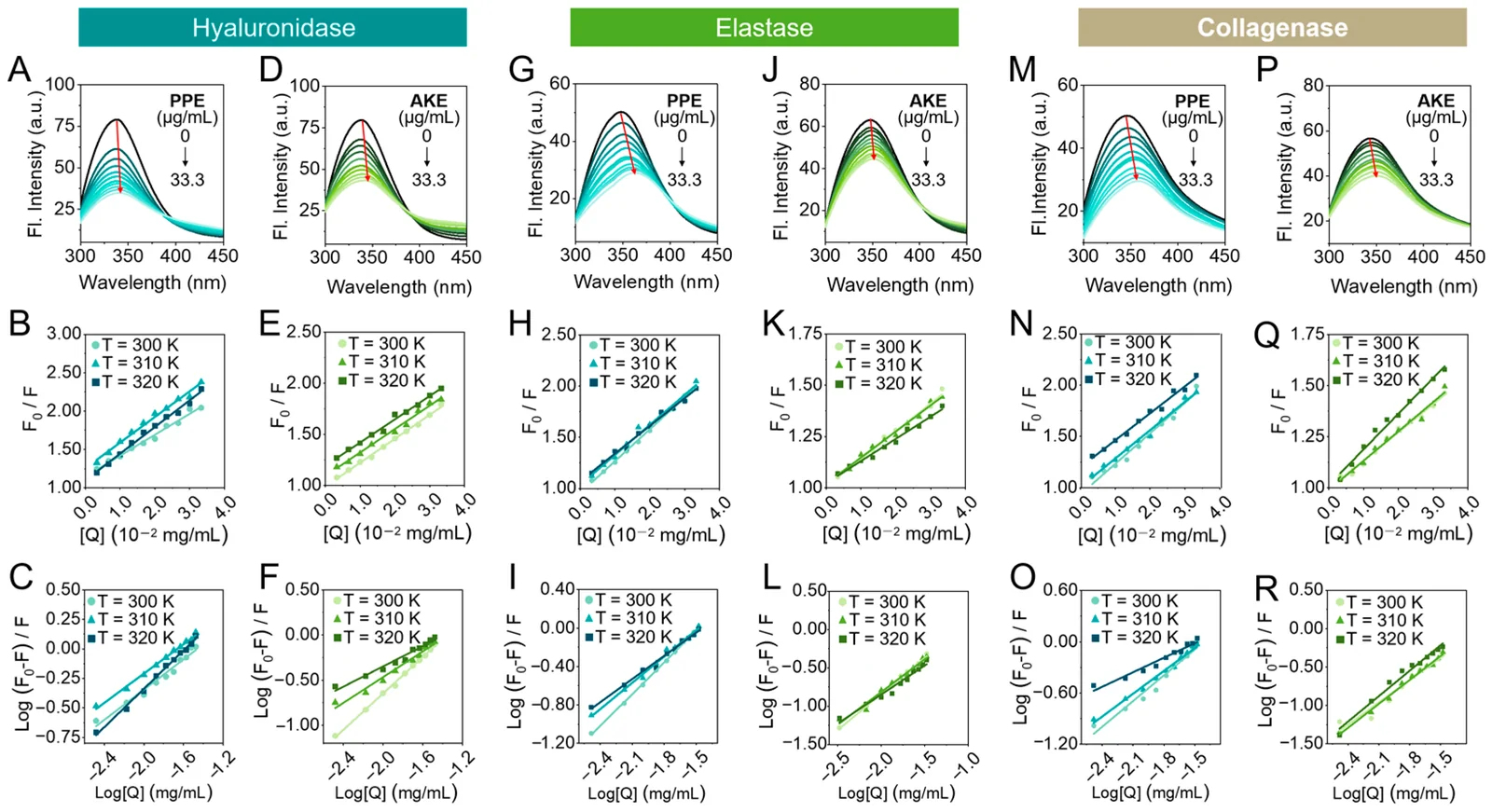
(**A**–**C**): Fluorescence emission spectra of HAase treated with increasing concentrations of **PPE** at 310 K (**A**); Stern–Volmer plots (**B**) and double-logarithmic Stern–Volmer plots (**C**) for fluorescence quenching of HAase by **PPE** at 300/310/320 K. (**D**–**F**): Fluorescence emission spectra of HAase treated with increasing concentrations of **AKE** at 310 K (**D**); Stern–Volmer plots (**E**) and double-logarithmic Stern–Volmer plots (**F**) for fluorescence quenching of HAase by **AKE** at 300/310/320 K. (**G**–**I**): Fluorescence emission spectra of elastase treated with increasing concentrations of **PPE** at 310 K (**G**); Stern–Volmer plots (**H**) and double-logarithmic Stern–Volmer plots (**I**) for fluorescence quenching of elastase by **PPE** at 300/310/320 K. (**J**–**L**): Fluorescence emission spectra of elastase treated with increasing concentrations of **AKE** at 310 K (**J**); Stern–Volmer plots (**K**) and double-logarithmic Stern–Volmer plots (**L**) for fluorescence quenching of elastase by **AKE** at 300/310/320 K. (**M**–**O**): Fluorescence emission spectra of collagenase treated with increasing concentrations of **PPE** at 310 K (**M**); Stern–Volmer plots (**N**) and double-logarithmic Stern–Volmer plots (**O**) for fluorescence quenching of collagenase by **PPE** at 300/310/320 K. (**P**–**R**): Fluorescence emission spectra of collagenase treated with increasing concentrations of **AKE** at 310 K (**P**); Stern–Volmer plots (**Q**) and double-logarithmic Stern–Volmer plots (**R**) for fluorescence quenching of collagenase by **AKE** at 300/310/320 K. All fluorescence measurements: λ_ex_ = 280 nm. Enzyme concentrations: HAase (160 U/mL), elastase (300 U/mL), collagenase (125 U/mL). **AKE**/**PPE** concentrations: 0, 3.3, 6.7, 10.0, 13.3, 16.7, 20.0, 23.3, 26.7, 30.0, and 33.3 μg/mL. Abbreviations: **AKE**, *Atractylodes macrocephala* Koidz extract; **PPE**, *Paeonia lactiflora* Pall. extract.

Fluorescence quenching spectroscopy can quantitatively analyze the interactions between molecules and provide information about the microenvironment. We performed fluorescence quenching measurements to investigate the binding behaviors of extracts against three aging-related cutaneous enzymes, including HAase, elastase and collagenase. Such enzymes possess endogenous fluorophore residues (tryptophan and tyrosine) that emit fluorescence at 340 nm when excited at 280 nm. Tryptophan acts as a reliable indicator to reflect microenvironment variations triggered by ligand binding or protein conformational changes [34,35]. The results are displayed in Figure 9. The fluorescence intensity of enzymes declined progressively with rising doses of extracts at 300, 310 and 320 K, accompanied by obvious red shifts in emission peaks. Such spectral shifts implied greater exposure of tryptophan residues to polar surroundings, which confirmed direct binding between the extracts and enzymes [36]. **AKE** produced prominent fluorescence quenching and emission shifts, indicative of tight binding to enzyme active pockets. These results further verified that **AKE** suppressed skin-degrading enzymes, which highlighted its efficacy as a natural bioactive agent preserving skin tissue balance. Supplementary fluorescence spectra obtained at 300 and 320 K are available in Figures S3–S5.

Fluorescence quenching may arise from molecular rearrangement, excited-state chemical reactions, or binding interactions between proteins and molecule ligands. Generally, it is categorized as two primary types: dynamic quenching driven by fluorophore–quencher collisions, and static quenching stemming from stable non-fluorescent ground-state complex formation [35]. The two quenching types display inverse temperature responses. Static quenching declines at elevated temperatures as ground-state complexes become unstable, while dynamic quenching strengthens with temperature growth accelerating more frequent intermolecular collisions [37]. To clarify the fluorescence quenching interaction between the extracts and each aging-related enzyme, the Stern–Volmer (*S-V*) model was adopted to compute the quenching constants (*K_sv_*). As illustrated in Figure 9, plots of *F*_0_*/F* against quencher concentration [*Q*] yielded linear Stern–Volmer curves for the three enzymes, with *K_sv_* constants obtained from curve slopes. All tested groups produced *K_q_* values featuring excellent linearity (R^2^ > 0.98), confirming the rationality of the *S-V* quenching model. The *K_sv_* constants for extracts interacting with HAase at 300, 310 and 320 K are shown in Table 1, and relevant results for elastase and collagenase are available in Supplementary Materials Tables S3 and S4. These results revealed that *K_sv_* constants of **PPE**–HAase and **AKE**–collagenase complexes increased as the temperature increased, consistent with the characteristic pattern of dynamic quenching. Conversely, the remaining complexes displayed declining *K_sv_* values under higher thermal conditions, confirming static quenching as the dominant interaction mode.

#### 2.10.1. Quantification of Binding Parameters and Characterization of Binding Sites

For small-molecule ligands binding separately to identical binding pockets of a macromolecule, the equilibrium distribution of free and bound molecules can be described by the double-logarithmic binding equation: log$\left[\frac{\left(F0-F\right)}{F}\right]\;$= log*Ka* + *n*log[*Q*]. In this formula, *K_a_* refers to the binding constant, while *n* denotes the number of binding sites. Linear fitting of log$\left[\frac{\left(F0-F\right)}{F}\right]$ versus log[*Q*] gives a straight curve, with the slope reflecting *n* and the vertical intercept equal to log*Ka* [38] (Figure 9). Table 1 and Supplementary Tables S3 and S4 summarize the binding constants (*K_a_*) and binding site numbers (*n*) determined at 300, 310 and 320 K. Further analysis of the results confirmed that *K_a_* values of **PPE**–HAase and **AKE**–collagenase complexes increased as temperature increased, which demonstrated stronger binding interactions between extracts and target enzymes under thermal conditions. On the contrary, other extract–enzyme complexes presented reverse temperature-dependent trends. The trend of *K_a_* values was highly consistent with those of *K_sv_*, which further validated our deduction of quenching modes. In addition, the values of binding site number *n* stayed near 1 across 300–320 K, demonstrating that both extracts interact with a single inhibitory pocket on each aging-related enzyme.

#### 2.10.2. Analysis of Thermodynamic Parameters and Binding Forces

Non-covalent interactions between bioactive compounds and proteins consist of hydrophobic effects, hydrogen bonds, van der Waals forces, and electrostatic interactions [36,37]. The signs and magnitudes of thermodynamic parameters (*∆G*, *∆H*, *∆S*) can be used to determine the predominant binding forces between proteins and ligands. According to previous reports [39,40], the type of intermolecular forces can be distinguished based on thermodynamic values of *∆H* and *∆S*: positive *∆H* and positive *∆S* indicate hydrophobic interaction-dominated binding; positive *∆H* and negative *∆S* correspond to electrostatic attraction as the major driving force; negative *∆H* and positive *∆S* suggest combined contributions from hydrophobic and electrostatic effects; negative *∆H* and negative *∆S* reflect dominant van der Waals forces and hydrogen bonds. Values of *∆H* and *∆S* were derived from the slope and y-intercept of linear plots of ln*K_a_* against 1/T (Figures S6 and S7). All thermodynamic constants for the extract–enzyme complex are listed in Table 1 and Supplementary Tables S3 and S4. All measured *∆G* values remained negative across the three tested temperatures, proving that the binding between each extract and its target enzyme proceeded spontaneously. The **AKE**–collagenase and **PPE**–HAase complexes displayed positive *ΔH* and *ΔS*, confirming hydrophobic forces as the primary driving factor for binding. From a thermodynamic perspective, a positive *ΔS* typically points to hydrophobic interaction: when small-molecule ligands attach to proteins, hydrophobic amino acid residues are shielded, and trapped water molecules are freed, resulting in increased system entropy. By contrast, the remaining extract–enzyme complexes yielded negative *ΔH* and *ΔS*, which indicated that van der Waals forces and hydrogen bonds constituted the major intermolecular driving forces. While individual noncovalent contacts are weak and lack specificity, the collective effect of multiple such interactions is capable of altering the conformation and biological function of target proteins [36]. The results demonstrated that the extracts are capable of binding to and regulating skin-degrading enzymes, which facilitates the preservation of skin homeostasis.

### 2.11. Antidermatophytic Activity Assessment

*M. gypseum* is a clinically important pathogenic dermatophyte commonly responsible for cutaneous fungal infections and related inflammatory responses, rendering it an ideal model for evaluating the antimycotic potential of natural extracts [23,41]. In this study, the in vitro antifungal activities of **AKE** and **PPE** against *M. gypseum* were assessed, and their growth inhibition rates and minimum inhibitory concentrations (MICs) were quantified. The results demonstrated that the growth of *M. gypseum* was markedly suppressed by the tested extracts (Tables S1 and S2). Specifically, **AKE** exhibited significantly superior antifungal efficacy compared with **PPE**. **AKE** achieved an inhibition rate of 65.653 ± 1.735% and an MIC value of 92.357 ± 1.032 μg/mL, whereas **PPE** showed only 33.032 ± 0.21% inhibition. Furthermore, **AKE** displayed typical dose-dependent antifungal activity, indicating that it had great potential for development as an alternative antifungal agent for the skin.

### 2.12. Pilot Study of Extracts on Skin Barrier Repair

**Figure 10:**
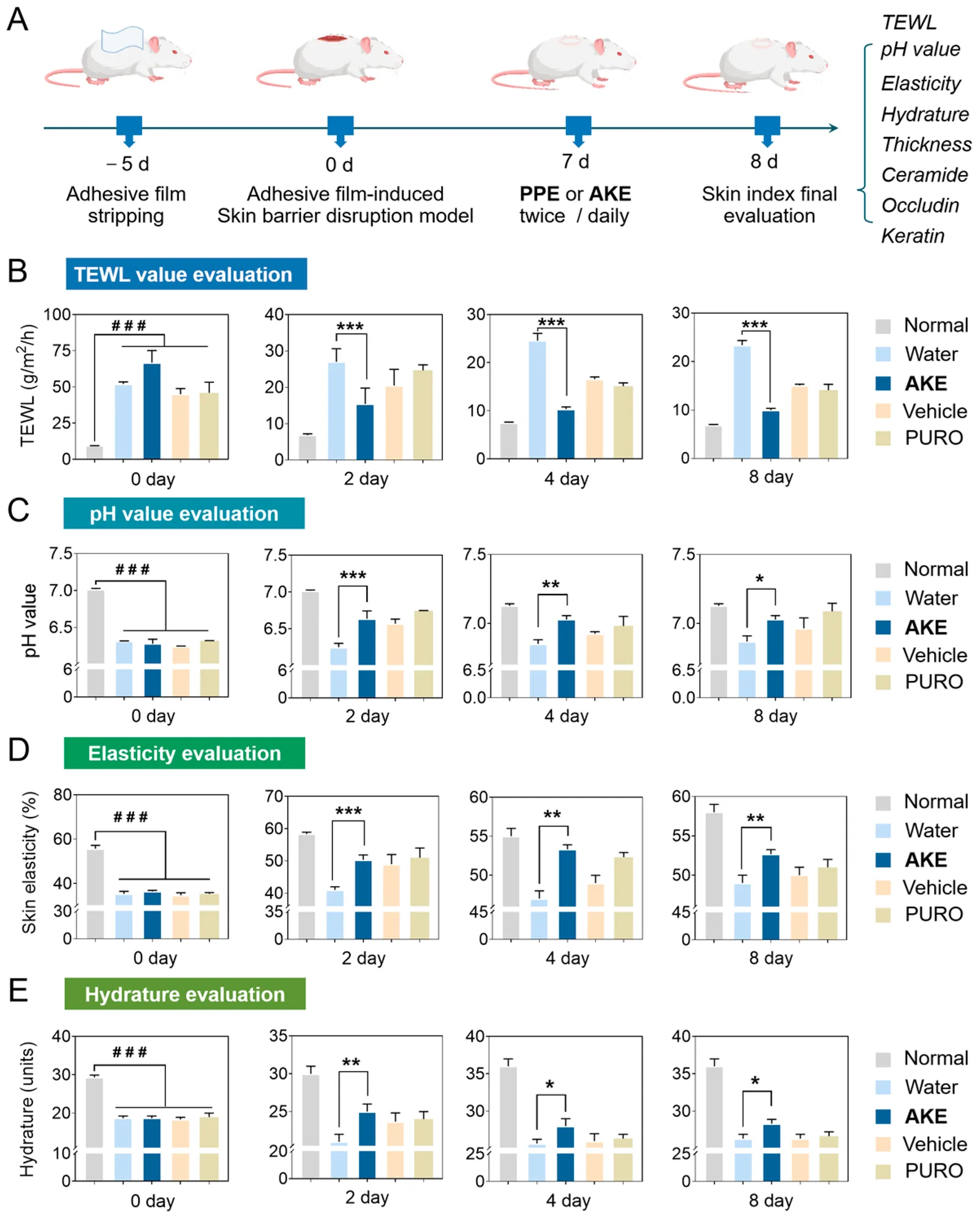
(**A**) Experimental process design. (**B**–**E**): Measurement results of TEWL (**B**), skin pH (**C**), elasticity (**D**), and hydration (**E**) at days 0, 2, 4, 8 after treatment. ^###^ *p* < 0.001, compared with the normal group; * *p* < 0.05, ** *p* < 0.01, and *** *p* < 0.001, compared with the model group. Abbreviations: **AKE**, *Atractylodes macrocephala* Koidz extract; **PPE**, *Paeonia lactiflora* Pall. extract; PURO, *Prinsepia utilis* Royle oil; TEWL, transepidermal water loss.

To assess the restorative efficacy of the extracts against physical epidermal damage, repeated tape stripping was carried out on mouse dorsal skin. The tape stripping protocol used herein only generated superficial mechanical damage limited to the stratum corneum instead of a chronic dermatitis model. Without continuous injury stimulation, the damaged phenotype in mice cannot be maintained in the long term. Accordingly, an 8-day evaluation time frame was selected to prevent confounding factors from compromising the reliability of index detection. Topical treatment with creams formulated with 10% extracts was administered, while PURO was set as the positive control group. Biophysical skin indicators such as TEWL, surface pH, elasticity and hydration were quantified on days 0, 2, 4 and 8 (Figure 10A). TEWL describes passive water evaporation originating from the inner skin across the epidermal layer. As a vital biophysical marker for skin barrier integrity, elevated TEWL readings directly reflect aggravated impairment of the skin barrier [18]. As presented in Figure 10B, treatment with extract cream induced a remarkable reduction in TEWL, demonstrating enhanced repair of damaged epidermal barriers. Notably, skin treated with **AKE** achieved a recovery rate of 81.2%, which was markedly superior to **PPE** (79.3%) and positive reference PURO (56.4%) after 8 days of topical administration. Additional biophysical skin metrics (pH, elasticity and hydration) were also significantly improved and recovered close to normal baseline levels (Figure 10C–E and Figure S8).

**Figure 11:**
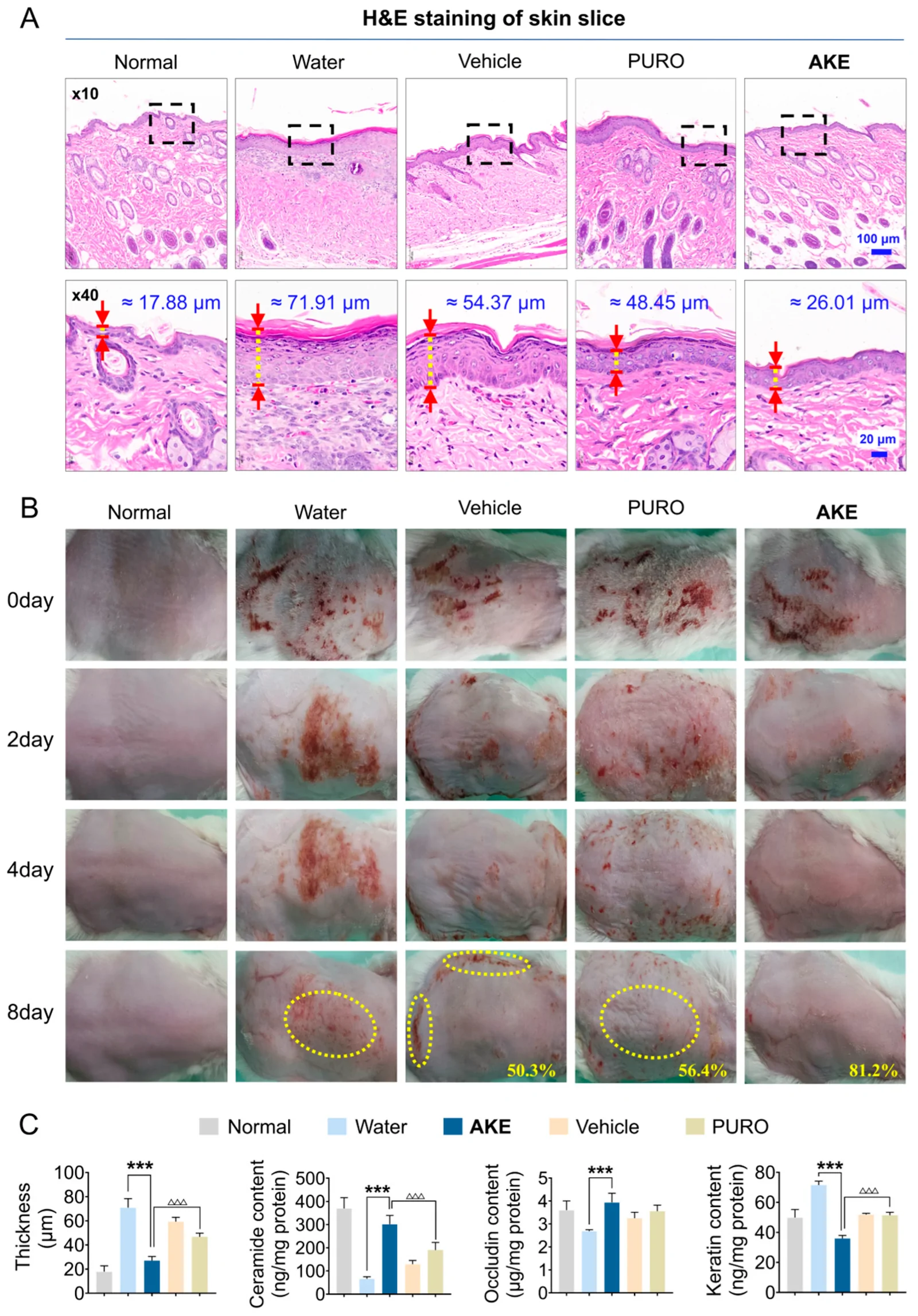
(**A**) Evaluation of skin tissue histopathology by H&E staining; the black curved box indicates the magnified region; red arrows indicate epidermal thickness measurement. (**B**) Representative images of mouse dorsal skin collected on days 0, 2, 4, and 8 post-treatment; yellow dashed circles mark visible skin damage regions. (**C**) Quantification of epidermal thickness, ceramide, as well as occludin and keratin levels in dorsal mouse skin. *** *p* < 0.001, compared with the model group. ^△△△^ *p* < 0.001, compared with the control reference group. Abbreviations: **AKE**, *Atractylodes macrocephala* Koidz extract; PURO, *Prinsepia utilis* Royle oil.

Based on skin appearance and histological staining results, repetitive tape stripping induced apparent redness, swelling and substantial damage to the epidermal barrier in murine dorsal skin. After an 8-day treatment with **AKE**, skin injuries were remarkably alleviated with diminished erythema. The skin thickness was reduced from 71.91 μm to 26.01 μm in the **AKE** group, which exhibited better efficacy than commercial reference PURO (48.45 μm) (Figure 11 and Figure S9). Furthermore, to explore the molecular regulatory mechanisms behind barrier repair, we quantified the expression of ceramide, as well as occludin and keratin levels, in dorsal skin tissues collected from the mice. As displayed in Figure 11C, the levels of ceramides and occludin were significantly upregulated to nearly healthy ranges after **AKE** treatment (*p* < 0.001), accompanied by prominent inhibition of abnormal keratin expression. Surprisingly, the regulatory effects of **AKE** were even superior to those of the commercial control reference group PURO (*p* < 0.001). These results substantiated the prominent capacity of **AKE** to facilitate skin barrier repair. Taken together, **AKE** facilitated epidermal barrier restoration via elevating ceramide and occludin contents and inhibiting keratin overproduction, which relieved epidermal hyperkeratosis and further relieved skin sensitivity symptoms. Collectively, these results confirmed that **AKE** possesses great prospects as a multi-functional ingredient to restore compromised epidermal barriers.

In the study, the promising epidermal repair and barrier protective efficacy of **AKE** was validated in the present mouse model, but several limitations of this study should be acknowledged to guide subsequent mechanistic exploration and translational application. First, the sample number of experimental mice adopted in the animal experiments was relatively limited, which may cause potential statistical bias and restrict the comprehensiveness and accuracy of the efficacy evaluation. Subsequent studies are required to expand the animal sample number for repeated verification and quantitative analysis, or further conduct human volunteer trials, so as to obtain more clinically credible results to validate the skin-protective effects of **AKE**. Second, the current in vivo experiments only focused on the local skin repair efficacy of topical **AKE** administration, without collecting mouse plasma and major organ tissues for subsequent biochemical and histological detection. Therefore, the systemic biodistribution, potential metabolic changes and distant organ responses after topical **AKE** application remain unclear, which limits the comprehensive biosafety assessment of **AKE**. Further systematic in vivo investigations are necessary to explore the systemic biological reactions induced by topical **AKE** treatment and clarify its biosafety profile, providing sufficient scientific evidence for the safe application and industrial transformation of **AKE** in skin repair and cosmetic fields. Third, changes in skin microbial composition and diversity upon **AKE** intervention were not evaluated in this study. Skin microbiota homeostasis is critically involved in sustaining epidermal barrier integrity and modulating skin inflammatory status. Without microbiome profiling, it remains unclear whether **AKE**-mediated skin barrier restoration is accompanied by remodeling of the beneficial skin microflora. Incorporating skin microbiome analysis in future work will help elucidate the microbial regulatory mechanism of **AKE**, enabling a more systematic and multidimensional evaluation of its beneficial skin functions.

### 2.13. LC-MS/MS Analysis of Extract Constituents

**Figure 12:**
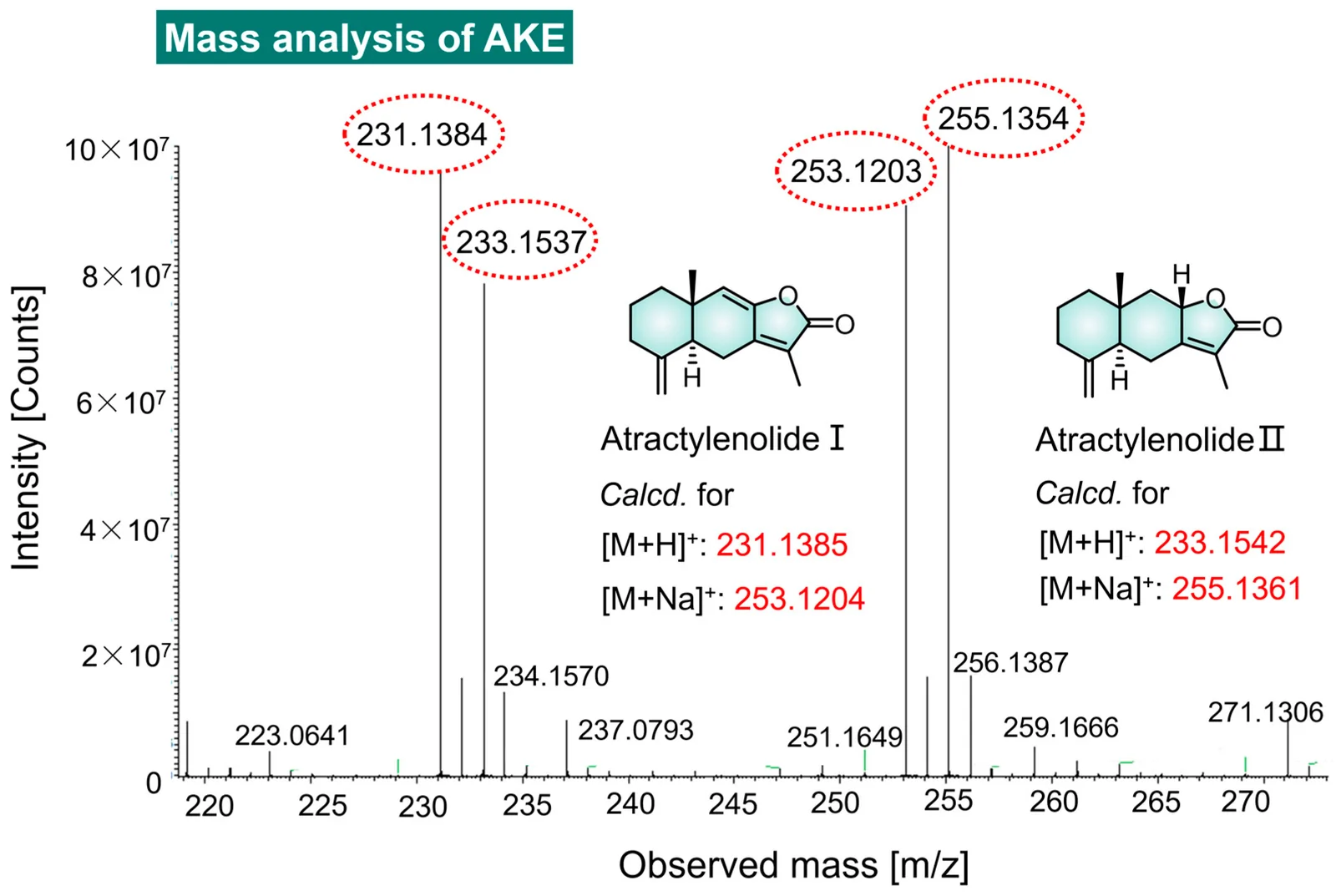
Structural characterization of the major compounds in **AKE** using LC-MS/MS technology. Abbreviations: **AKE**, *Atractylodes macrocephala* Koidz extract. The red circled peaks represent the experimentally observed quasi-molecular ion peaks.

LC-MS/MS analysis is a prevalent analytical method for determining phytochemical components in herbal extracts, as it offers outstanding reliability and sensitivity to acquire retention times, precise mass values and fragment ions [42]. UHPLC-ESI-HRMS/MS in positive ionization mode was utilized to characterize candidate bioactive molecules within **AKE**. The fragment ion peaks are illustrated in Figure 12. Signals at *m*/*z* 231.1384 [M+H]^+^ and *m*/*z* 253.1203 [M + Na]^+^ were assigned to atractylenolide I in **AKE**, while peaks at *m*/*z* 233.1537 [M + H]^+^ and *m*/*z* 255.1354 [M + Na]^+^ corresponded to the molecular profile of atractylenolide II. Chemical constituents were identified by matching accurate mass data (mass deviation ≤ 5 ppm) against previously published literature [43]. By integrating LC-MS identification results with their potent anti-inflammatory properties, atractylenolide I and atractylenolide II were proposed as the potential bioactive components driving epidermal barrier repair. Similar mass spectrometry analysis of **PPE** uncovered paeoniflorin and albiflorin as its major bioactive constituents (Figure S10).

### 2.14. Molecular Docking

**Figure 13:**
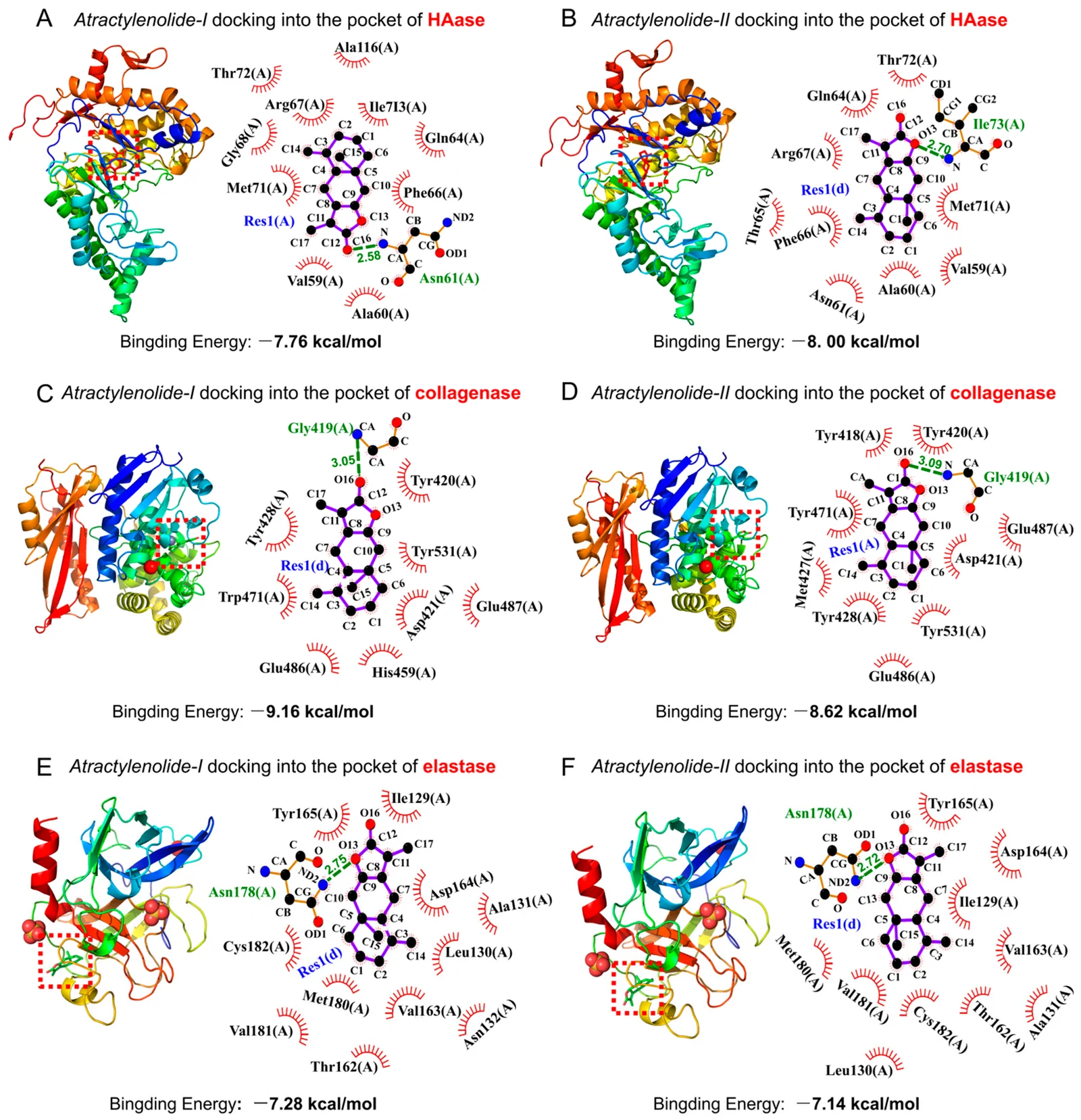
Molecular docking of Atractylenolide-I and Atractylenolide-II against HAase, collagenase and elastase. (**A**,**B**) Binding modes of Atractylenolide-I and Atractylenolide-II in the active pocket of HAase. (**C**,**D**) Docking poses of Atractylenolide-I and Atractylenolide-II within collagenase pocket. (**E**,**F**) Complex conformations of Atractylenolide-I and Atractylenolide-II with elastase. Left: the 3D ligand–protein structures. Right: 2D interaction diagrams showing hydrogen bond. In the docking diagrams, red dashed squares indicate ligand binding sites; green dashed lines indicate the hydrogen bonds; and semicircular arcs correspond to hydrophobic contacts. Abbreviations: HAase, hyaluronidase.

Molecular docking analysis is an effective approach to predict binding modes between ligand inhibitors and protein receptors, which can quantify binding affinity and clarify intermolecular interaction mechanisms [42,44]. We carried out molecular docking against three skin-degrading enzymes (HAase, elastase and collagenase) to verify the bioactive roles of major bioactive in extracts (Figure 13). Figure 13A,B reveal that atractylenolide I and II both form stable binding complexes with HAase. Multiple hydrogen bonds and hydrophobic contacts were detected between the two ligands and key amino acid residues, namely Tyr202(A), Trp130(A), Ala116(A), Val59(A), Trp42(A), Val322(A), Thr72(A), and Asn61(A). In general, lower binding energies indicate tighter intermolecular interactions between ligands and proteins [42]. Atractylenolide I (−7.76 kcal/mol) and atractylenolide II (−8.00 kcal/mol) displayed higher binding affinity for HAase than paeoniflorin (−5.81 kcal/mol) and albiflorin (−5.40 kcal/mol) (Figure S11A,B). Parallel docking analysis was conducted for collagenase and elastase (Figure 13C–F and Figure S11C–F). Collectively, the results of these molecular docking studies corroborated the conclusions of the preceding skin degrading enzyme inhibitory assays (Figure 5B–D). Atractylenolide I and II were potential bioactive components of **AKE** that exhibited significant inhibitory activity against these enzymes. The identification of these bioactive molecules also lays the foundation for studying the molecular mechanisms by which **AKE** repairs the epidermal barrier.

## 3. Materials and Methods

### 3.1. Materials and Instruments

All solvents and chemical reagents applied in this work were of analytical purity. Specifically, the enhanced Cell Counting Kit-8 (CCK-8, Cat. No. C0042), BCA protein assay kit (Cat. No. P0010), NO assay kit (Cat. No. S0021S), and bovine serum albumin (BSA, purity > 98%) were supplied by Beyotime Biotechnology (Shanghai, China). Lipopolysaccharide (LPS) was acquired from TargetMol (Shanghai, China). Various ELISA kits were obtained from distinct suppliers. Kits targeting human ceramide (Cat. No. YJ540321), occludin (Cat. No. YJ010830), keratin (Cat. No. YJ329201), and murine keratin (Cat. No. JK99852) were obtained from Shanghai Enzyme-linked Biotechnology Co., Ltd. (Shanghai, China). ELISA kits for detecting mouse ceramide (Cat. No. KQ112032) and occludin (Cat. No. KQ111910) were purchased from Shanghai Keqiao Biotechnology Co., Ltd. (Shanghai, China). Assay kits for human IL-6 (Cat. No. YJ058097) and IL-8 (Cat. No. YJ46909) were obtained from Elabscience Biotechnology Co., Ltd. (Wuhan, China). In addition, elastase and the chromogenic substrate N-Succinyl-Ala-Ala-Ala-p-Nitroanilide (purity > 98%) were sourced from Beijing Aobosen Biotechnology Co., Ltd. (Beijing, China) and Shanghai Aladdin Biochemical Technology Co., Ltd. (Shanghai, China), respectively. The peptide N-[3-(2-furyl) acryloyl]-Leu-Gly-Pro-Ala, two formulations of Tris-HCl buffer (0.05 mol/L, pH 7.5; 1 mol/L, pH 8.0) and HAase were purchased from Shanghai Yuanye Biotechnology Co., Ltd. (Shanghai, China). Ursolic acid (≥98% purity), sodium hyaluronate (≥93% purity, MW = 1,800,000 Da), and Type I collagenase (125 U/mg, originating from *Clostridium histolyticum*) were supplied by Shanghai Titan Scientific Co., Ltd. (Shanghai, China). The dermatophyte *Microsporum gypseum* CBS118893 was obtained from the Medical Fungi Preservation Center of the Chinese Academy of Medical Sciences (Nanjing, China). Terbinafine hydrochloride and DMSO were sourced from Sigma-Aldrich (St. Louis, MO, USA), and agar powder together with chloramphenicol were acquired from dedicated scientific research reagent suppliers.

### 3.2. Plant Materials

Dried roots and rhizomes of four medicinal herbs, namely *D. officinale*, *P. odoratum*, *P. lactiflora* and *A. macrocephala*, were obtained from the herbal market of Luosiwan International Trade City (Kunming, China) in April 2023. The raw materials were collected in September 2022 from three production areas: Yulong County (Lijiang), Malipo County (Wenshan) and Tengchong County (Baoshan). Dr. Ying Zhou from the School of Chemical Science and Technology, Yunnan University, verified the taxonomic identities of all samples. Corresponding voucher specimens with serial codes YNU20230402–YNU20230405 were archived in the Laboratory of Intelligent Molecular Synthesis and Functionalization at Yunnan University. Figure S1 shows the procedure for *P. lactiflora*: 300 g of dried root and rhizome material was subjected to reflux extraction with 80% aqueous ethanol (3 × 3.0 L, 3 h cycles) maintained at 60 °C. The extracts were evaporated by rotary evaporation to produce a target fraction (75.8 g), which was then resuspended in water and partitioned against petroleum ether, followed by n-butanol. The extract **PPE** (50.2 g) was obtained after concentration of n-butanol fraction. As depicted in Figure S1, 300 g of *A. macrocephala* powder was soaked in ethyl acetate and processed via ultrasonic-assisted extraction at room temperature for three 24 h cycles, each using 3 L of solvent. Removing the solvent in vacuo delivered the **AKE** extract (60.1 g). Ultimately, the **AKE** and **PPE** extracts were subsequently used for further research.

### 3.3. Cell Culture

The human HaCaT keratinocyte cell line was supplied by Meisen CTCC (Zhejiang Meisen Cell Technology Co., Ltd., Jinhua, China), while RAW264.7 macrophage cells were purchased from Fuheng Biotechnology Co., Ltd. (Shanghai, China). Both cell lines were cultured in DMEM containing 10% fetal bovine serum (FBS) and 1% penicillin–streptomycin solution (purchased from Zhejiang Meisen Cell Technology Co., Ltd.). Cell cultivation was performed at 37 °C in a humidified atmosphere with 5% CO_2_ until cells entered the logarithmic growth stage.

### 3.4. Cytotoxicity Assay

CCK-8 assays were performed to evaluate the cytotoxic potential of the tested extracts. Briefly, HaCaT cells were inoculated into 96-well plates at a density of 1 × 10^5^ cells per well and cultured overnight. The cells were then exposed to serially diluted extracts for 24, 48 or 72 h, with five parallel replicates for each treatment group. Subsequently, 10 μL CCK-8 reagent was supplemented to each well and incubated at 37 °C for 2 h. The optical density was recorded at 450 nm using a microplate spectrophotometer (Shanghai Flash Spectrum Biological Technology Co., Ltd., Shanghai, China) [12].

### 3.5. Nitric Oxide Inhibition Activity Measure

The anti-inflammatory ability of the extract was assessed through NO inhibitory assays. RAW264.7 macrophages were plated in 24-well plates at 8.0 × 10^4^ cells/well and incubated for 24 h at 37 °C under 5% CO_2_ atmosphere. The cells were co-treated with 2 μg/mL LPS and extracts at 25 μg/mL. After a further 24 h incubation, supernatants were collected by centrifugation, and NO concentrations were determined following the standard operating procedures of the NO detection kits (Beyotime Biotechnology, Shanghai, China, Cat. No. S0021S).

### 3.6. Wound-Healing Assay

The wound-healing assay was performed with minor adjustments referenced from published work [24]. Briefly, HaCaT cells were seeded into 12-well plates at a density of 3 × 10^5^ cells/mL and incubated overnight to form confluent monolayers. Linear scratches were made across the cell layer using a 20 μL pipette tip, and loose cells were rinsed off with sterile PBS. Cells were then cultured in serum-free medium supplemented with 50 μg/mL extracts. Bright-field images of the scratched areas were captured at 10× magnification via a Ruihoge microscope at 0 h and 24 h after scratching. The residual wound area was quantified via ImageJ-Fiji software (version 1.54p), and wound closure percentages were calculated by the equation below:

Wound closure area (%) = 100% − [(OWA_24h_/OWA_0h_) × 100%]

In this formula, OWA_24h_ refers to the residual wound area measured at 24 h post-treatment, and OWA_0h_ stands for the initial wound area captured right after scratching.

### 3.7. Detection of ROS Production

The levels of intracellular ROS were measured using ROS Assay Kit with DCFH-DA (Beyotime Biotechnology, Shanghai, China, Cat. No. S1105S). HaCaT cells were pretreated with extracts and/or SLS for 24 h and incubated with 10 µM DCFH-DA for 25 min at 37 °C. The cells were observed under a fluorescence microscope (Leica Fluorescence Confocal, Wetzlar, Germany).

### 3.8. Enzyme-Linked Immunosorbent Assay

Once HaCaT cells grew to approximately 80% confluence, different doses of extracts (6.25–50 μg/mL) as well as positive control were administered to the cells. After 24 h treatment, supernatants were collected by centrifugation, and the concentrations of IL-6 and IL-8 were measured by ELISA following the official kit protocols. To detect intracellular functional proteins, HaCaT cells were incubated with extracts (6.25–50 μg/mL) for 24 h prior to cell lysis in RIPA buffer. The BCA method was used for protein quantification, and ELISA kits were utilized to test the contents of ceramide, occludin and keratin. The above protocol was also carried out to investigate the time-related effects at 24, 48 and 72 h.

### 3.9. Evaluation of Skin-Related Enzyme Inhibitory Activity

#### 3.9.1. Anti-Hyaluronidase Assay

In this study, skin-related enzyme inhibition assays were performed with commercially purified enzymes in a cell-free in vitro biochemical system. The HAase suppressive capacity of samples was determined by measuring the yield of N-acetyl-glucosamine degraded from sodium hyaluronate, according to published protocols [45,46]. Briefly, 30 μL sample (0.125–5.00 mg/mL), 30 μL HAase (10 U/mL) and 10 μL phosphate buffer (300 mM, pH 5.35) were mixed and incubated at 37 °C in sequence. Then 30 μL hyaluronic acid substrate (0.03%) was supplemented to start the reaction, which lasted for 45 min. Afterwards, 100 μL acid albumin solution (0.1% BSA, pH 3.75) was added to stop the reaction, and the system was incubated for another 10 min at 37 °C. Absorbance was recorded at 600 nm, with ursolic acid set as the positive control group. The inhibitory percentage was calculated using the following formula:

Enzyme inhibition activity (%) = [(OD_sample_ − OD_control_)/OD_sample_] ×100%

#### 3.9.2. Anti-Elastase Assay

Elastase inhibitory activity was determined following a previously described protocol [47]. Briefly, 50 μL sample (0.125–5.00 mg/mL), 50 μL elastase (0.5 U/mL) and 50 μL Tris-HCl buffer (0.1 mM, pH 8.0) were blended and incubated at 37 °C for 15 min. The chromogenic substrate N-Succinyl-Ala-Ala-Ala-p-nitroanilide (1 mM, 50 μL) was subsequently added, and the reaction proceeded for 20 min. Absorbance was measured at 410 nm. Ursolic acid was adopted as the positive control, and 0.5% DMSO was used as the vehicle control.

#### 3.9.3. Anti-Collagenase Assay

Collagenase inhibitory activity was determined via a spectrophotometric method reported previously [48]. Briefly, 50 μL collagenase (1 U/mL), 50 μL Tris-HCl buffer (0.05 mM, pH 7.5) and 50 μL sample (0.125–5.00 mg/mL) were mixed and incubated at 37 °C for 15 min. The chromogenic substrate N-[3-(2-furyl) acryloyl]-Leu-Gly-Pro-Ala (0.48 mg/mL, 50 μL) was then added, followed by a 20 min reaction period. Absorbance was tested at 335 nm. Ursolic acid served as the positive control, while 0.5% DMSO was set as the vehicle control.

### 3.10. Western Blot

Western blot analysis was conducted to assess the levels of epidermal barrier and inflammatory proteins in HaCaT cells. Cells were divided into five experimental groups, including blank control group, SLS-induced injury model group, **AKE** treatment group, **PPE** treatment group, and positive control (PURO) group. For intervention groups, cells were pretreated with 50 μg/mL extracts before exposure to 20 μg/mL SLS. The treated cells were harvested and lysed in RIPA buffer containing protease inhibitor cocktail. The cell lysate was quantified using the BCA protein assay kit (Beyotime, China). About 10 μg of total proteins was applied to SDS/PAGE, and then transferred to polyvinylidene difluoride (PVDF) membrane. After blocking in 5% non-fat milk in TBST for 1 h at room temperature, membranes were incubated with primary antibodies overnight at 4 °C, then incubated with horseradish peroxidase-labeled secondary anti bodies and visualized with ECL. Protein bands’ grayscale densities were quantified with ImageJ. Total protein expression was normalized to GAPDH, whereas phosphorylated protein levels were normalized to their respective total protein.

### 3.11. Fluorescence Quenching Studies

#### 3.11.1. Fluorescence Quenching of Three Skin-Degrading Enzymes

Fluorometric titration was carried out to quantitatively characterize the binding interactions between extracts and three skin-degrading enzymes. Stock enzyme solutions (3.0 mL) were formulated at 160 U/mL (HAase), 300 U/mL (elastase) and 125 U/mL (collagenase), respectively. Gradual titration with 5 μL solutions of 2.0 mg/mL extract stock was performed for each enzyme solution, generating final extract concentrations between 0 and 33.3 μg/mL. Following each titration addition, the mixture was incubated at 300 K, 310 K and 320 K for 5 min, and fluorescence emission spectra were subsequently collected via a spectrofluorometer. The excitation wavelength was fixed at 280 nm, with emission scanning ranging from 300 nm to 500 nm. Excitation and emission slits were adjusted to 10 nm, and the scan rate was set to 3000 nm/min. A circulating water bath provided stable temperature conditions. The Stern–Volmer equation was applied to interpret the fluorescence quenching mechanism [35,36]:

(1) $$\frac{F_{0}}{F}=1+Kq\tau 0[Q]=1+Ksv[Q]$$

Equation (1) is defined as follows: *F*_0_ and *F* are the enzyme fluorescence intensities measured before and after quencher treatment, respectively; Kq is the biomolecular quenching rate constant; [*Q*] is the concentration of quencher, and *τ*0 corresponds to the average lifetime of biomolecules without quencher (a standard value of 10^−8^ s). *K_SV_* represents the Stern–Volmer dynamic quenching constant. Linear regression analysis of *F*_0_*/F* plotted against [*Q*] was used to calculate *K_SV_* at each tested temperature.

#### 3.11.2. Binding Constant and Binding Number

The double-logarithmic formula was adopted to determine the binding constant (*Ka*) and the number of binding sites (*n*):

(2) $$\log\frac{F_{0}-F}{F}=logKa+nlog[Q]$$

#### 3.11.3. Thermodynamic Characteristics of Interactions

Thermodynamic analysis helps identify the dominant intermolecular forces between the extract quencher and target enzymes. Since the association constant (*K_a_*) varies with temperature, thermodynamic calculations were performed to quantify the binding characteristics. The van ’t Hoff equation was applied to calculate core thermodynamic indices: enthalpy change (∆*H*), entropy change (∆*S*) and free energy change (∆*G*), as shown below:

(3) $$\log\mathrm{Ka}\;=\;-\frac{∆H}{2.303\mathrm{RT}}+\frac{∆S}{2.303R}$$

In this equation, *R* stands for the gas constant, while *T* refers to the experimental temperatures (300 K, 310 K and 320 K). Linear fitting of logK_a_ versus 1/T was conducted, and the corresponding slope and intercept were used to calculate ∆*H* and ∆S. The Gibbs free energy change (∆*G*) was determined via the formula below:

∆*G* = ∆*H* − *T*∆*S* (4)

### 3.12. Evaluation of the Anti-Dermatophytic Activity

A modified broth microdilution assay was adopted to evaluate the anti-dermatophyte effects of herbal extracts against *M. gypseum* in 96-well plates [41]. Samples were serially diluted, and fungal suspension was added to each well to yield an inoculum density of 5 × 10^5^ CFU/mL. After 5 days of incubation at 25 °C, the absorbance at 625 nm was recorded spectrophotometrically. Three control groups were included in parallel: blank medium, untreated fungal growth, and terbinafine hydrochloride as positive control. The Reed–Muench algorithm was applied to compute the minimum inhibitory concentration (MIC_50_) of all samples.

### 3.13. Pilot Study of the Repair Effect of the Skin Barrier in Mice

Six-week-old healthy male CD-1/ICR mice (18–22 g) were supplied by Hunan SJA Laboratory Animal Co., Ltd., Changsha, China. The mice were maintained in a constant environment at 24 ± 2 °C and 55 ± 10% humidity under a 12 h light–dark photoperiod. Standard laboratory diet and purified water were available to the mice ad libitum. All animal operations complied with the international guidelines for laboratory animal welfare. The animal protocol was reviewed and approved by Institutional Animal Care and Use Committee of Yunnan Luoyu Biotechnology Co., Ltd. (Kunming, China) (Approval Number: PZ20240403). Efforts were made to minimize animal suffering and to reduce the number of animals used. In total, 18 mice were randomly divided into six groups (*n* = 3 per group): normal control group, water-treated model group, vehicle cream group, 10% **PPE** cream group, 10% **AKE** cream group and positive control 10% PURO cream group. Prior to repetitive tape stripping, a 2 × 2 cm^2^ area was marked on the dorsal skin of each mouse. Long hairs were removed using an electric shaver, and residual hairs in this region were depilated with Veet cream for 2 min. The depilated dorsal area was then wiped with cotton swabs soaked in distilled water. All groups except the normal control underwent daily adhesive tape stripping for 5 days to disrupt the epidermal barrier, with successful modeling confirmed when TEWL exceeded 35 mg/cm^2^/h. The TEWL detection was performed using a Tewameter TM300 (Courage Khazaka, Cologne, Germany). Untreated normal mice served as the blank control, while the other five groups received twice-daily topical administration (200 μL per application) of distilled water, 10% PPE cream, 10% AKE cream, 10% PURO cream or vehicle cream base for 8 days. Skin biophysical indicators including TEWL, surface pH, elasticity and hydration were measured on days 0, 2, 4 and 8 to evaluate barrier restoration, and the skin recovery rate was computed via the formula shown below [18]:

$$\mathrm{Barrier}\;\mathrm{recovery}\;\mathrm{rate}\;=\;\frac{\mathrm{TEWL}(\mathrm{post}-\mathrm{stripping})-\mathrm{TEWL}(\mathrm{time point})}{\mathrm{TEWL}(\mathrm{post}-\mathrm{stripping})-\mathrm{TEWL}(\mathrm{baseline})}\times 100\%$$

where TEWL (post-stripping) represents TEWL values right after epidermal barrier damage; TEWL (time point) is TEWL measured at each detection time point; and TEW (baseline) refers to the initial TEWL value of intact skin.

The formula ratio of cream base is shown in Table S5.

### 3.14. Histological Examination

We carried out histological analysis of dorsal mouse skin following a previously reported protocol [18]. Following the measurement of skin-related parameters on day 8, experimental mice were euthanized, and dorsal skin tissues were immediately harvested for histological analysis and biochemical quantification. Each skin specimen was separated into two parts for morphological assessment and the other for biochemical quantification of ceramide, occludin, and keratin levels, respectively. For histological observation, tissue samples were fixed in 10% neutral buffered formalin, followed by paraffin embedding, and sliced into 6 μm thick sections. The sections were subjected to hematoxylin and eosin (H&E) staining to assess epidermal structure and pathological alterations. Epidermal thickness was quantified with a Pannoramic MID II scanner (3DHISTECH Ltd., Budapest, Hungary) to evaluate skin barrier restoration. Each sample was measured independently, and the acquired data were used for subsequent statistical analysis.

### 3.15. Identification of Constituents in Extracts by LC-MS/MS Analysis

Chromatographic separation was performed on a Hypersil GOLD Phenyl C4 column (100 mm × 2.1 mm, particle size 1.9 μm). A gradient elution program was applied with a mobile phase comprising 0.1% aqueous formic acid and methanol at a volume ratio of 2:98 throughout the 5 min run. An injection volume of 5 μL was used, and the flow rate was maintained at 0.3 mL/min. Mass spectrometric data were collected via an electrospray ionization (ESI) source operating in positive ion mode. The instrumental parameters were configured as follows: sheath gas flow rate, 13.5 L/min; auxiliary gas flow rate, 3 L/min; spray voltage, 3 kV; capillary temperature, 350 °C. The mass scan range was defined as *m*/*z* 80–1200.

### 3.16. Molecular Docking Simulation

Molecular docking was performed to explore the interactions between bioactive compounds (atractylenolide I, atractylenolide II, paeoniflorin, and albiflorin,) and three skin-degrading enzymes, namely HAase, elastase, and collagenase [49]. The three-dimensional (3D) structures of the three enzymes were retrieved from the Protein Data Bank (PDB, https://www.rcsb.org/): HAase (PDB ID: 2PE4), collagenase (PDB ID: 4AR1), and elastase (PDB ID: 3EST). Before molecular docking simulation, all protein structures were preprocessed via Visual Molecular Dynamics (VMD) software (version 1.9.3) to eliminate endogenous ligands and solvent molecules, so as to guarantee the structural integrity and uniformity of protein models [35,49]. The 3D structures of these compounds were geometrically optimized via energy minimization with Gaussian 09 software at the B3LYP/6-31G basis set level. Docking simulations were performed using AutoDock 4.2. The docking box was defined to cover the whole protein structure, enabling exhaustive screening of all potential binding pockets. Flexible docking was adopted to accommodate conformational rearrangements of both ligands and target proteins upon complex formation. Grid box parameters for each enzyme were configured as detailed below: for HAase, the grid center coordinates were x = 38.54, y = −25.778, z = −8.639, with grid dimensions of 126 Å along all three axes; for elastase, the grid center coordinates were x = −4.525, y = 28.35, z = 43.101; the grid dimensions were 108 Å for the *x*-axis, 90 Å for the *y*-axis, and 100 Å for the *z*-axis; and for collagenase, the grid center coordinates were x = −7.175, y = −29.506, z = 13.012, and the grid dimensions were 108 Å along all three axes.

### 3.17. Statistical Analysis

GraphPad Prism 8.0 software was utilized for all statistical analyses. Intergroup statistical comparisons were conducted via one-way analysis of variance (ANOVA). All quantitative data are presented as mean ± standard deviation (SD), with results obtained from no fewer than three independent biological replicates. Differences were regarded as statistically significant when *p* < 0.05.

## 4. Conclusions

Natural herbal extracts are one of the ideal sources for developing gentle barrier repair ingredients. Previous studies on *A. macrocephala* have mainly focused on its gastrointestinal and immunomodulatory properties; its skin repair applications remain underexamined. In the present study, we comparatively assessed the skin barrier protective performance of **AKE** and **PPE**, and demonstrated that **AKE** possessed superior multi-pathway efficacy to repair impaired epidermal homeostasis by a series of experiments, bridging the existing research gap in this area. At the cellular level, **AKE** promoted HaCaT keratinocyte migration, alleviated oxidative injury induced by SLS, and significantly upregulated intracellular ceramide biosynthesis, which was indispensable for reconstructing the intercellular matrix of stratum corneum. Biochemical enzyme assays confirmed that **AKE** suppressed the catalytic activities of HAase, collagenase and elastase, three important hydrolases responsible for extracellular matrix degradation, skin dryness and cutaneous aging. Fluorescence quenching spectroscopy coupled with thermodynamic calculations substantiated spontaneous non-covalent binding between **AKE** components and these skin degrading enzymes, offering a molecular explanation for its enzyme inhibitory effects. With respect to inflammatory regulation, **AKE** markedly reduced the production of pro-inflammatory cytokines IL-6 and IL-8, restored the levels of tight junction protein occludin, normalized dysregulated keratin level, and inhibited activation of the NF-κB signaling pathway in damaged keratinocytes. In addition, **AKE** exhibited inhibitory activity against the pathogenic dermatophyte *M. gypseum* with an MIC value of 92.357 ± 1.032 μg/mL, implying its potential for relieving dermatomycosis-linked pruritus and inflammation. Consistent with in vitro observations, topical **AKE** application achieved an 81.2% epidermal integrity recovery rate in tape-stripped mice, outperforming **PPE** and commercial ingredient PURO. **AKE** intervention effectively reversed abnormal alterations of key skin biophysical indicators, including TEWL, surface pH, elasticity and stratum corneum hydration. Histopathological analysis confirmed that the treatment effectively reduced epidermal hyperplasia, while simultaneously normalizing tissue concentrations of ceramide and occludin. Subsequent UHPLC-ESI-HRMS/MS analysis and molecular docking simulation identified the potential contribution of atractylenolide I and atractylenolide II. Taken together, our findings illustrated that **AKE** repaired the impaired skin barrier through multi-target regulation of lipid synthesis, ROS scavenging, inhibition of skin degrading enzymes, stabilization of tight junction proteins, suppression of NF-κB-mediated inflammation and anti-dermatophyte activities. This multi-target regulation highlighted that **AKE** is a functional ingredient with the potential to maintain the integrity of the epidermal barrier and alleviate symptoms of sensitive skin. Future work is needed to isolate individual pure compounds from this extract and elucidate their underlying molecular mechanisms, which will facilitate its practical translation and application.

## Figures and Tables

**Table 1:** Quenching constants (*K_SV_*), binding constants (*K_a_*) for HAase–**AKE** and HAase–**PPE** interactions at varying temperatures.

Sample	Temp. (K)	*K*_sv_ (mL/mg)	*K*_a_ (mL/mg)	*n*	∆*S* (J/mol·K)	∆*H* (KJ/mol)	∆*G* (KJ/mol)
**AKE**	300	23.4	2321.9	1.007	−68.232	−23.793	−3.323
	310	23.0	902.7	0.712			−2.641
	320	22.8	591.4	0.560			−1.959
**PPE**	300	27.3	867.0	0.642	124.338	32.070	−5.232
	310	33.4	1084.7	0.622			−6.475
	320	34.8	1944.5	0.814			−7.719

## Data Availability

The data that support the findings of this study are available from the corresponding author upon reasonable request.

## Supplementary Materials

The following supporting information can be downloaded at: https://www.mdpi.com/article/10.3390/molecules31203713/s1.

## Abbreviations

The following abbreviations are used in this manuscript:

**AKE**	*Atractylodes macrocephala* Koidz extracts
**PPE**	*Paeonia lactiflora* Pall. extracts
TEWL	Transepidermal water loss
NO	Nitric oxide
HaCaT	Human keratinocytes
HAase	Hyaluronidase
PURO	*Prinsepia utilis* Royle oil
*A. macrocephala*	*Atractylodes macrocephala* Koidz.
*P. lactiflora*	*Paeonia lactiflora* Pall.
SLS	Sodium lauryl sulfate
DCFH-DA	2′,7′-Dichlorodihydrofluorescein diacetate.
ROS	Reactive oxygen species
ECM	Extracellular matrix
TJ	Tight junction
IL	Interleukin
ELISA	Enzyme linked immunosorbent assay
SPTLC1	Serine palmitoyltransferase long chain-1
*S-V*	Stern-Volmer
Ksv	Quenching constants
Ka	Binding constant
N	Number of binding sites
*M. gypseum*	*Microsporum gypseum*
MIC	Minimum inhibitory concentrations
CCK-8	Cell Counting Kit-8
