# Supplementary material for: Evaluation of Atractylodes macrocephala Koidz Extract as a Multi-Functional Natural Ingredient for Skin Barrier Repair
Source: Molecules. 2026 Oct 16;31(20):3713. doi: 10.3390/molecules31203713 (PMC13707995; doi:10.3390/molecules31203713)
Supplement: Supplementary file 1 [file molecules-31-03713-s001.zip › molecules-4550795-supplementary.pdf]

Supporting Information

Evaluation of *Atractylodes macrocephala* Koidz Extract as a Multi-Functional Natural Ingredient for Skin Barrier Repair

Huan Liu [1, †], Le Yu [1, †], Chang-Yin Yang [2], Yu-Qiang Zhao [1], Lin Xu [2,], Ying Zhou [1,*]*

1 Yunnan Characteristic Plant Extraction Laboratory, School of Chemical Science and Technology, Yunnan University, Kunming 650091, China.

2 Liwa Institute of Skin Health, School of Chemistry and Molecular Engineering, East China Normal University, Shanghai 200062, China

* Correspondence: yingzhou@ynu.edu.cn

† The authors contribute equally to this work.

Supplemental Figures and Tables

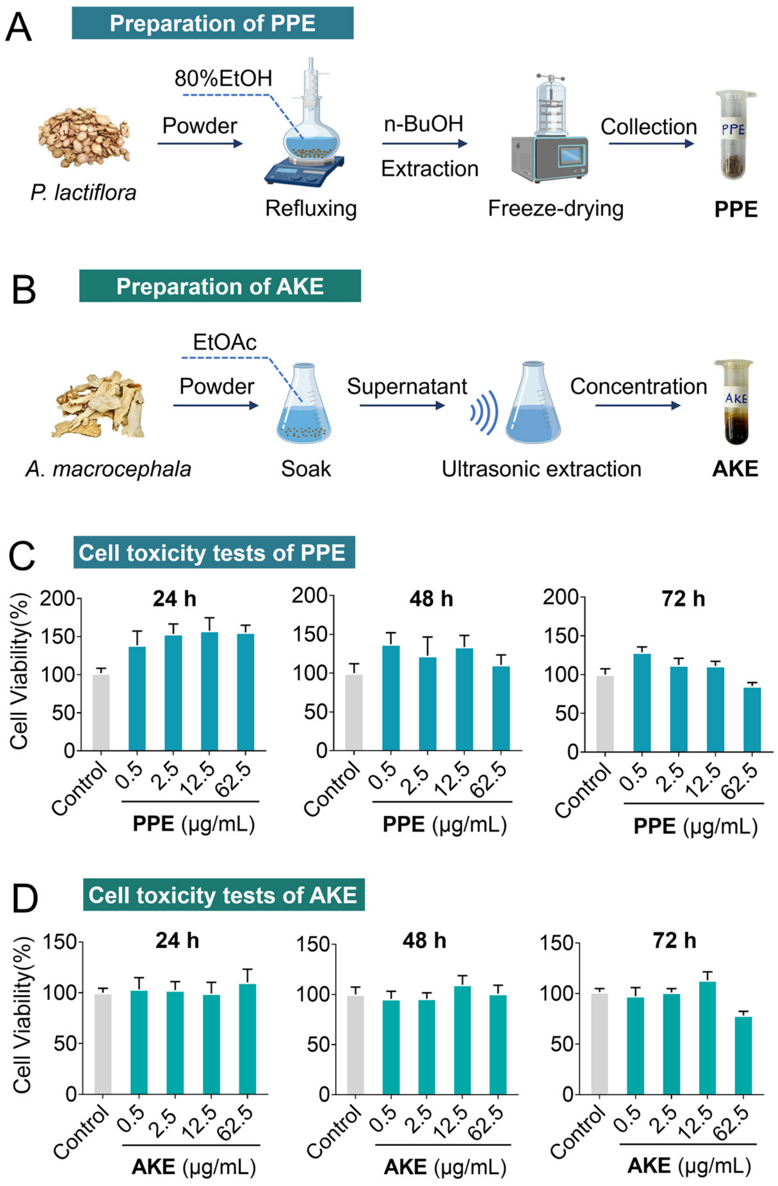

Figure S1. (A-B) Preparation process of **AKE** and **PPE**. (C-D) Effects of different concentrations of **AKE** (C) and **PPE** (D) on the viability of HaCaT cells after 24, 48, and 72 hours of treatment. Abbreviations: **AKE**, *Atractylodes macrocephala* Koidz extract; **PPE**, *Paeonia lactiflora* Pall. extract.

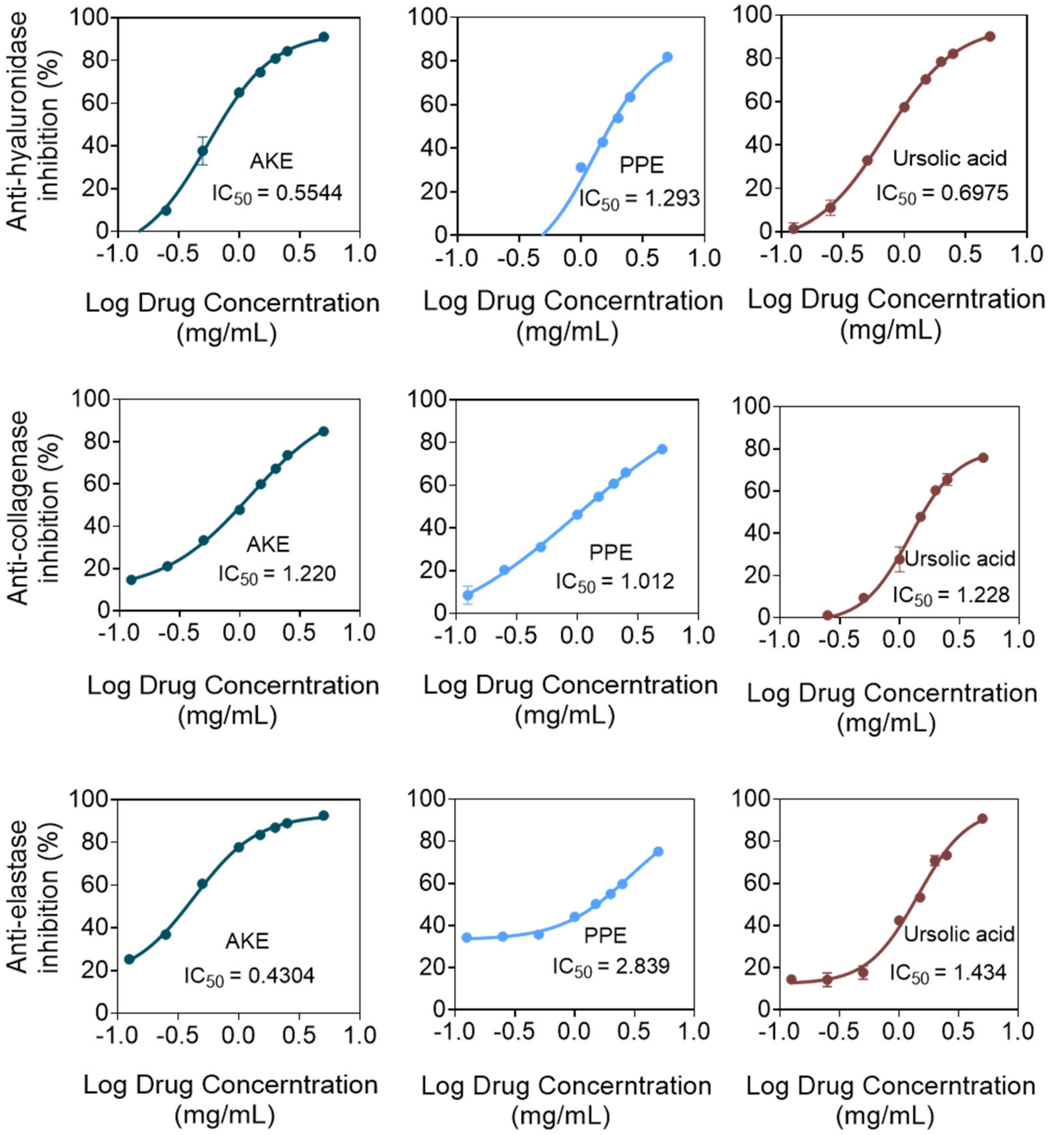

Figure S2. IC_{50} value hyaluronidase, elastase and collagenase inhibitory activity of **PPE** and **AKE.** Abbreviations: **AKE**, *Atractylodes macrocephala* Koidz extract; **PPE**, *Paeonia lactiflora* Pall. extract.

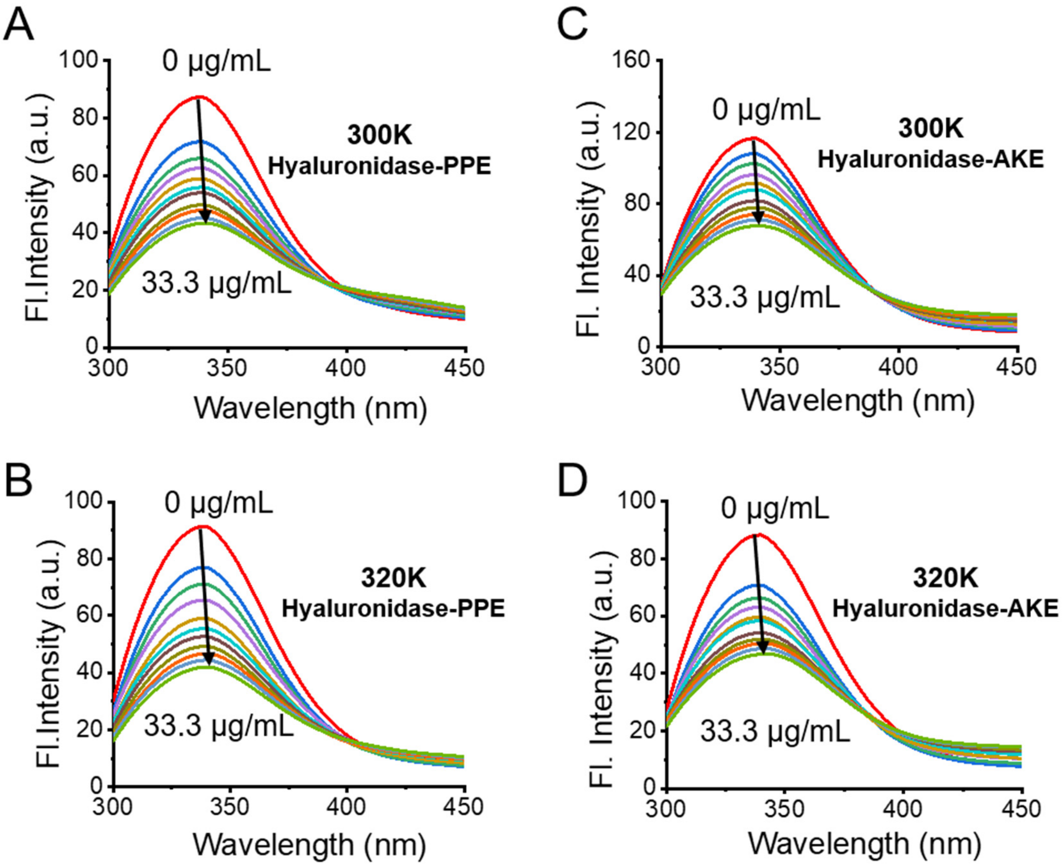

Figure S3. Fluorescence emission spectra of hyaluronidase in the presence of different concentrations of **PPE** (A) and **AKE** (B) at 300 K, 320 K. (Ex = 280 nm, c(hyaluronidase) = 160 U/mL and c (**PPE**/**AKE**) = 0,3.3, 6.7, 10.0, 13.3, 16.7, 20.0, 23.3, 26.7, 30.0, 33.3 μg/mL). Abbreviations: **AKE**, *Atractylodes macrocephala* Koidz extract; **PPE**, *Paeonia lactiflora* Pall. extract.

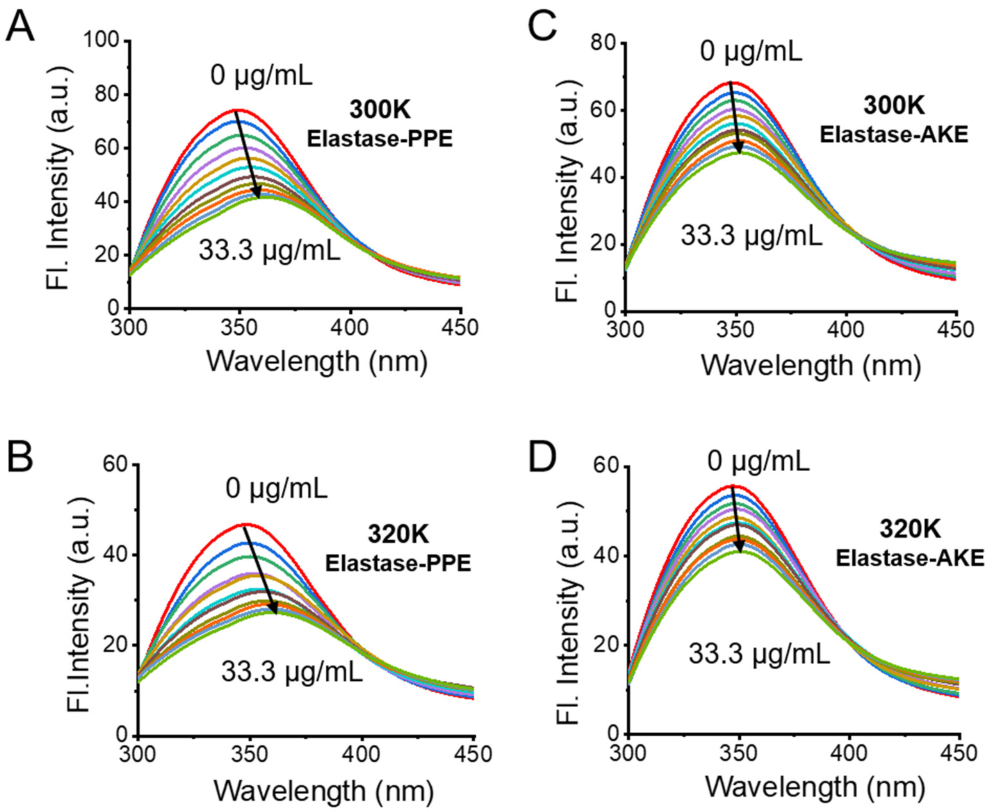

Figure S4. Fluorescence emission spectra of elastase in the presence of different concentrations of **PPE** (A) and **AKE** (B) at 300 K, 320 K. (Ex = 280 nm, c(elastase) = 300 U/mL and c (**PPE**/**AKE**) = 0,3.3, 6.7, 10.0, 13.3, 16.7, 20.0, 23.3, 26.7, 30.0, 33.3 μg/mL). Abbreviations: **AKE**, *Atractylodes macrocephala* Koidz extract; **PPE**, *Paeonia lactiflora* Pall. extract.

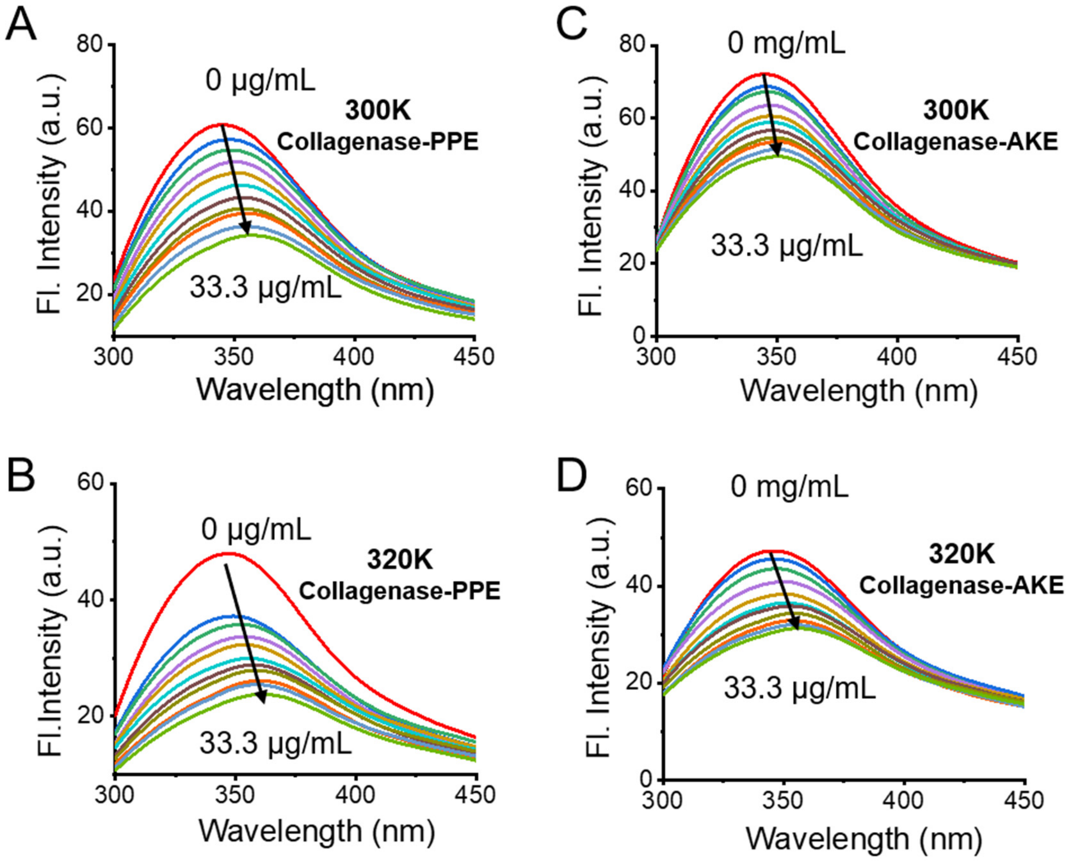

Figure S5. Fluorescence emission spectra of collagenase in the presence of different concentrations of **PPE** (A) and **AKE** (B) at 300 K, 320 K. (Ex = 280 nm, c(collagenase) = 125 U/mL and c (**PPE**/**AKE**) = 0,3.3, 6.7, 10.0, 13.3, 16.7, 20.0, 23.3, 26.7, 30.0, 33.3 μg/mL). Abbreviations: **AKE**, *Atractylodes macrocephala* Koidz extract; **PPE**, *Paeonia lactiflora* Pall. extract.

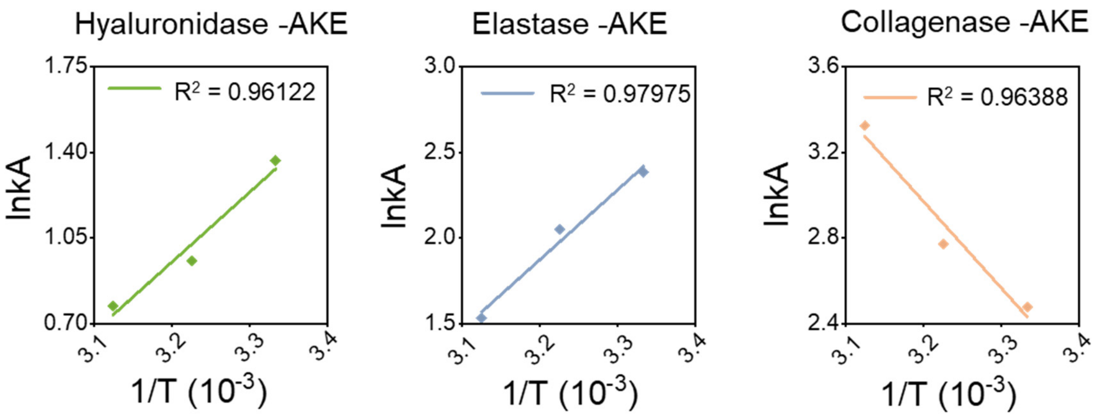

Figure S6. The Vant't Hoff plots for the fluorescence quenching of hyaluronidase, elastase, collagenase by **AKE.** Abbreviations: **AKE**, *Atractylodes macrocephala Koidz* extract.

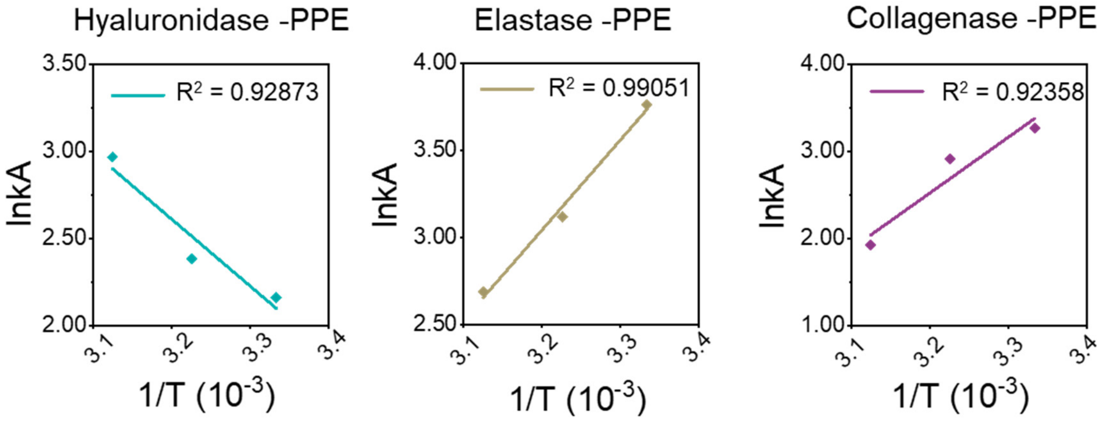

Figure S7. The Vant't Hoff plots for the fluorescence quenching of hyaluronidase, elastase, collagenase by **PPE**. Abbreviations: **AKE**, *Atractylodes macrocephala* Koidz extract.

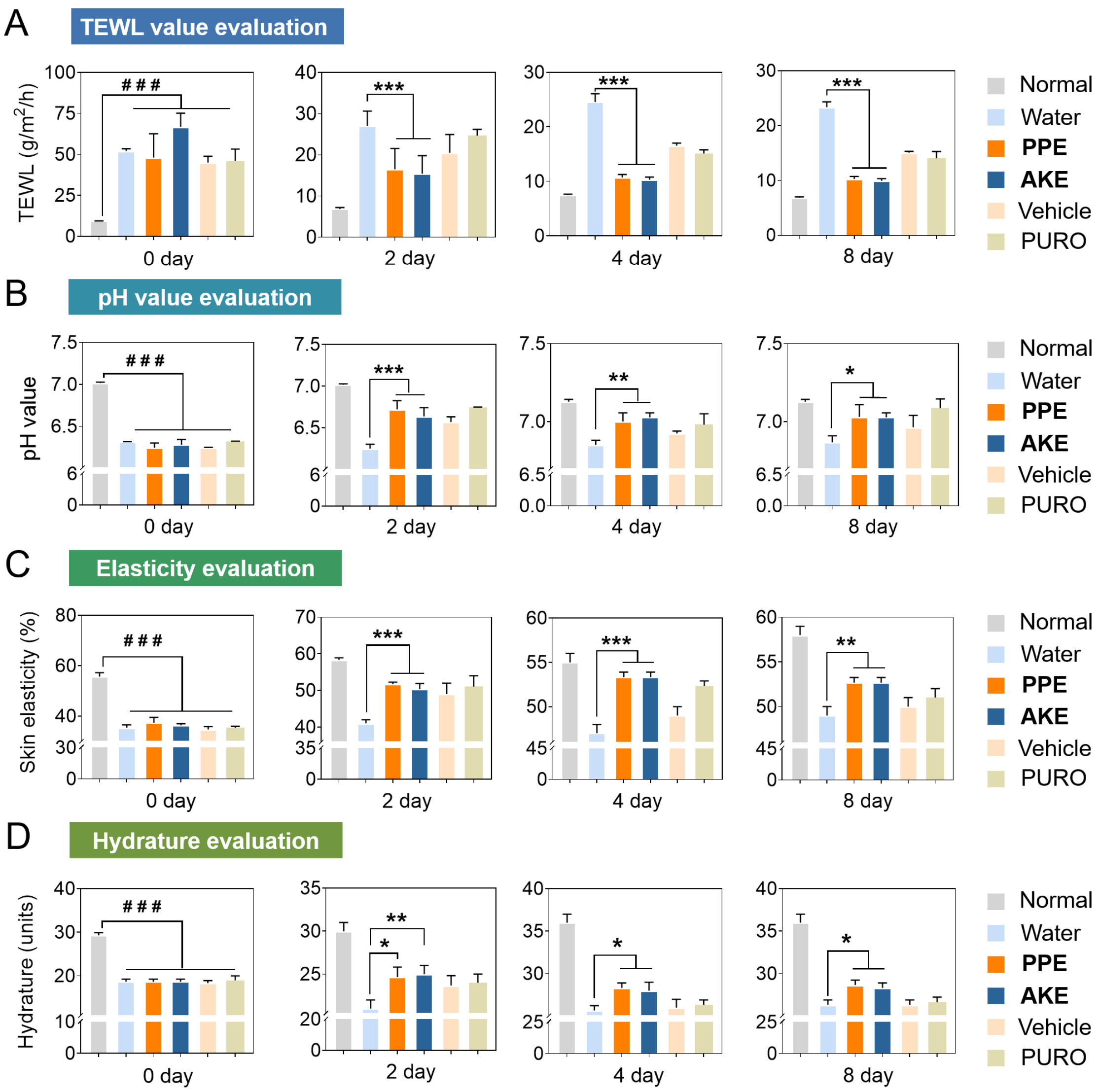

Figure S8. (A–D): Comparison of TEWL (A), skin pH (B), elasticity (C), and hydration (D) measured at days 0, 2, 4, and 8 following treatments. ### $p < 0.001$, compared with the normal group; * $p < 0.05$, ** $p < 0.01$, and *** $p < 0.001$, compared with the model group. Abbreviations: **AKE**, *Atractylodes macrocephala* Koidz extract; **PPE**, *Paeonia lactiflora* Pall. extract; PURO, *Prinsepia utilis* Royle oil; TEWL, transepidermal water loss.

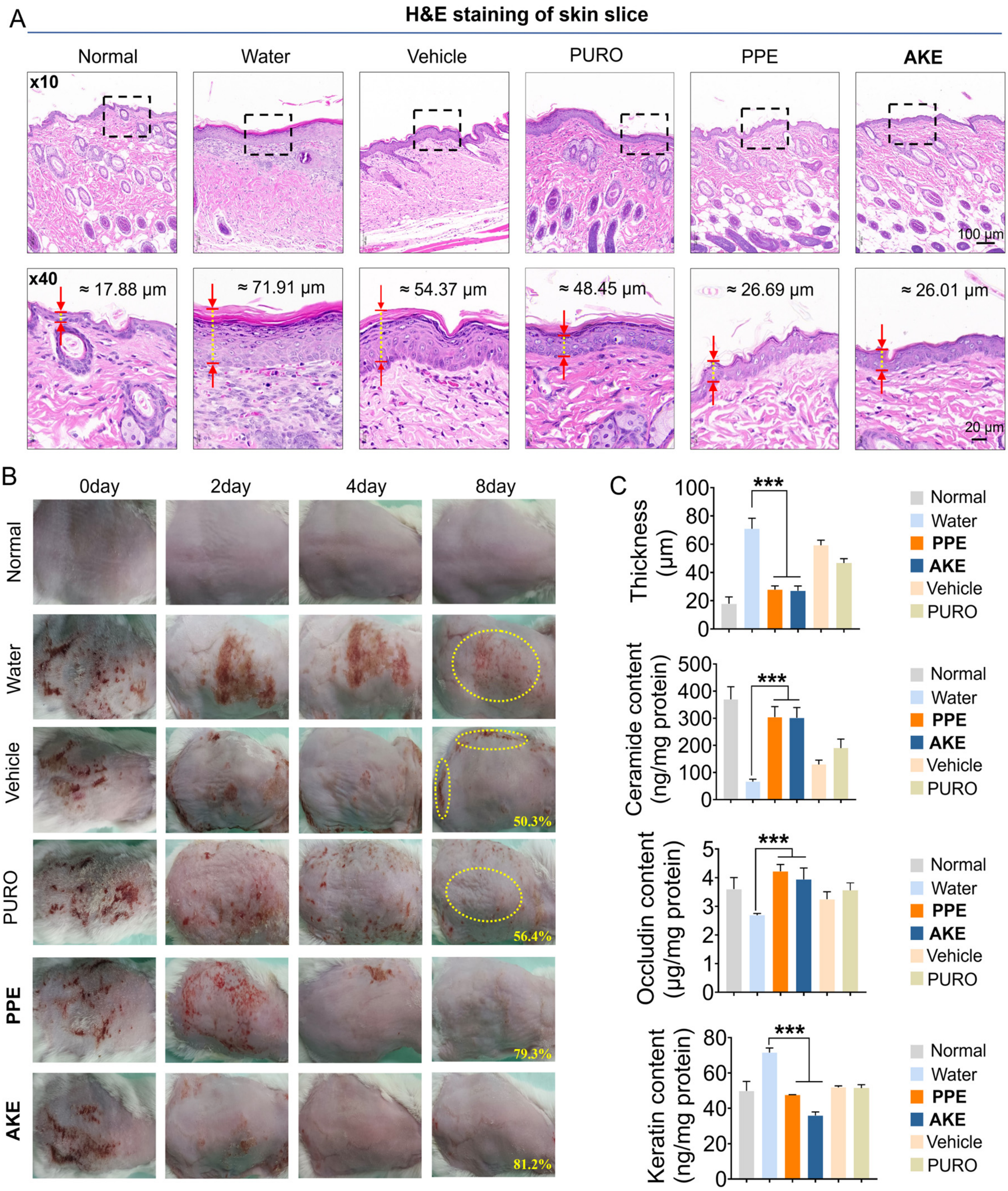

Figure S9. (A) Histological analysis of skin lesions using hematoxylin and eosin (H&E) staining. (B) Representative images of dorsal skin from mice on days 0, 2, 4, and 8 following treatments. (C) Comparison of epidermal thickness, as well as ceramide, occludin, and keratin content in the dorsal skin of mice across different treatment groups. *** $p < 0.001$, compared with the model group. Abbreviations: **AKE**, *Atractylodes macrocephala* Koidz extract; **PPE**, *Paeonia lactiflora* Pall. extract; PURO, *Prinsepia utilis* Royle oil. The black curved box indicates the magnified region; red arrows indicate epidermal thickness measurement; yellow dashed circles mark visible skin damage regions.

Mass analysis of PPE

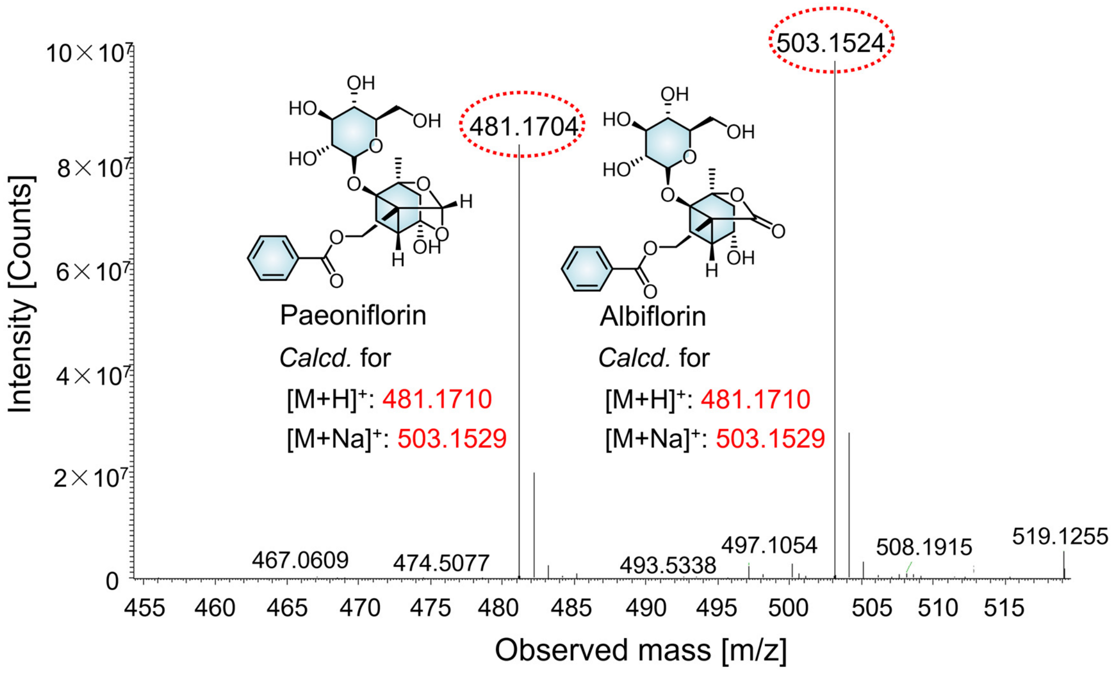

Figure S10. The LC-MS/MS analysis results about the chemical structures of principal components identified in **PPE**. Abbreviations: **PPE**, *Paeonia lactiflora* Pall. extract.

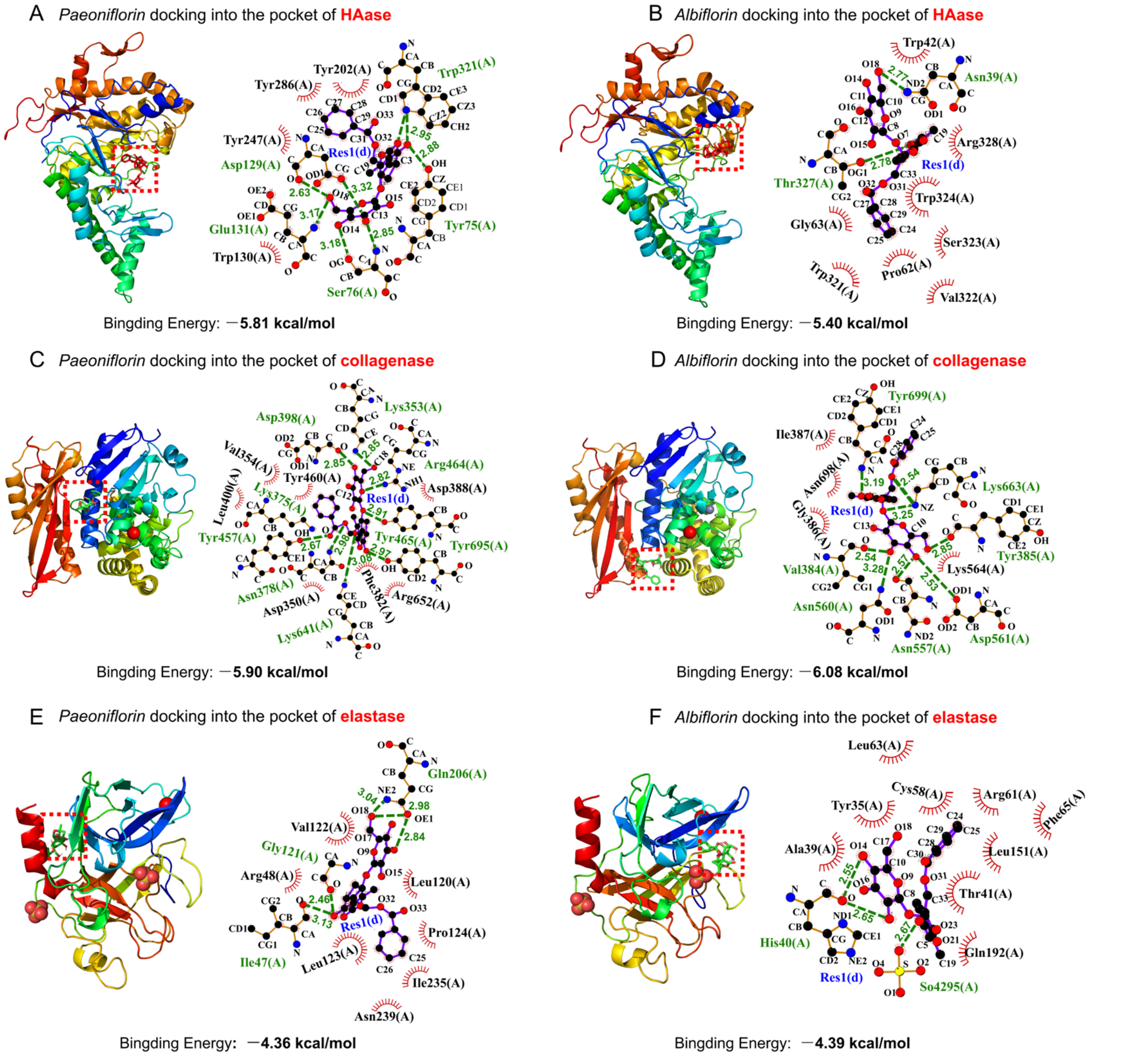

Figure S11. Molecular docking of Paeoniflorin and Albiflorin against HAase, collagenase and elastase. (A-B) Binding modes of Paeoniflorin and Albiflorin in the active pocket of HAase. (C-D) Docking poses of Paeoniflorin and Albiflorin within collagenase pocket. (E-F) Complex conformations of Paeoniflorin and Albiflorin with elastase. Left: the 3D ligand-protein structures; Right: 2D interaction diagrams showing hydrogen bond. In the docking diagrams, red dashed squares indicate ligand binding sites; green dashed lines indicate the hydrogen bonds, and semicircular arcs correspond to hydrophobic contacts. Abbreviations: HAase, hyaluronidase.

Table S1. Antidermatophytic activity of **AKE** and **PPE** against *M. gypseuma.*

Sample	Concentration	Inhibition（%）
Terbinafine Hydrochloride	1 μg/mL	99.013±0.855
**PPE**	128 μg/mL	33.032±0.21
**AKE**	128 μg/mL	65.653±1.735

Table S2. Antidermatophytic activity of **AKE** against *M. gypseuma*.

**Sample**	**Concentration**	**Inhibition（%）**	**MIC$_{50}$**
Terbinafine Hydrochloride (ng/mL)	200	103.801±0.452	0.717±0.009
	40	103.681±0.539	
	8	101.287±1.528	
	1.6	77.107±3.048	
	0.32	22.881±2.286	
**AKE** （μg/mL）	100	53.291±0.748	92.357±1.032
	50	22.019±0.42	
	25	19.05±3.779	
	12.5	16.824±3.569	
	6.25	7.769±3.359	

Table S3. Quenching constants (K_{SV}), binding constants (K_a) and relative thermodynamic parameters of the interaction between **AKE**/**PPE** and elastase at different temperatures.

Sample	Temp. (K)	K_{sv} (mL/mg)	K_a (mL/mg)	n	ΔS (J/mol·K)	ΔH (KJ/mol)	ΔG (KJ/mol)
	300	31.1	4314.1	1.107			−9.316
**PPE**	310	29.3	2255.6	0.916	−112.720	−43.132	−8.188
	320	27.1	1466.8	0.808			−7.061
	300	13.5	1085.5	0.938			−6.033
**AKE**	310	12.7	777.2	0.853	−92.986	−33.929	−5.103
	320	10.8	462.8	0.757			−4.173

Table S4. Quenching constants (K_{SV}), binding constants (K_a) and relative thermodynamic parameters of the interaction between **AKE**/**PPE** and collagenase at different temperatures.

Sample	Temp. (K)	K_{sv} (mL/mg)	K_a (mL/mg)	n	ΔS (J/mol·K）	ΔH (KJ/mol)	ΔG (KJ/mol)
	300	29.8	2632.2	1.007			−8.425
**PPE**	310	27.7	1834.2	0.892	−150.073	−53.448	−6.925
	320	26.3	684.8	0.568			−5.424
	300	13.8	1191.5	0.970			−6.066
**AKE**	310	14.3	1601.2	1.040	132.455	33.670	−7.391
	320	17.4	2778.6	1.106			−8.715

Table S5. The formula ratio of cream base.

Trade Name	INCI Name (English)	CAS Number	Content (%)
Tego Care 165	Glyceryl Stearate<br>PEG-100 Stearate	123-94-4<br>9004-99-3	2
IMEX MCT 60/40	Caprylic / Capric Triglyceride	65381-09-1,73398-61-5	15
Water	Water (Aqua)	7732-18-5	80.3
Euxyl PE 9010	Phenoxyethanol<br>Ethylhexylglycerin	122-99-6<br>70445-33-9	0.5
Aristoflex AVC	Ammonium Acryloyldimethyltaurate / VP Copolymer		0.2
Emercol C16-18 30:70 MY	Cetearyl Alcohol	67762-27-0 / 8005-44-5	2
		total	100
